# TiO_2_ protective layers shape photocathode performance in photoelectrochemical CO_2_ reduction

**DOI:** 10.1093/nsr/nwag092

**Published:** 2026-02-09

**Authors:** Linxiao Wu, Yumeng Han, Hao Chen, Peixuan Liu, Jinshui Cheng, Bin Shao, Jingshan Luo

**Affiliations:** Institute of Photoelectronic Thin Film Devices and Technology, State Key Laboratory of Photovoltaic Materials and Cells, Tianjin Key Laboratory of Efficient Solar Energy Utilization, Ministry of Education Engineering Research Center of Thin Film Photoelectronic Technology, Nankai University, Tianjin 300350, China; Institute of Photoelectronic Thin Film Devices and Technology, State Key Laboratory of Photovoltaic Materials and Cells, Tianjin Key Laboratory of Efficient Solar Energy Utilization, Ministry of Education Engineering Research Center of Thin Film Photoelectronic Technology, Nankai University, Tianjin 300350, China; Institute of Photoelectronic Thin Film Devices and Technology, State Key Laboratory of Photovoltaic Materials and Cells, Tianjin Key Laboratory of Efficient Solar Energy Utilization, Ministry of Education Engineering Research Center of Thin Film Photoelectronic Technology, Nankai University, Tianjin 300350, China; College of Electronic Information and Optical Engineering, Tianjin Key Laboratory of Optoelectronic Sensor and Sensing Network Technology, Nankai University, Tianjin 300350, China; Institute of Photoelectronic Thin Film Devices and Technology, State Key Laboratory of Photovoltaic Materials and Cells, Tianjin Key Laboratory of Efficient Solar Energy Utilization, Ministry of Education Engineering Research Center of Thin Film Photoelectronic Technology, Nankai University, Tianjin 300350, China; College of Electronic Information and Optical Engineering, Tianjin Key Laboratory of Optoelectronic Sensor and Sensing Network Technology, Nankai University, Tianjin 300350, China; Institute of Photoelectronic Thin Film Devices and Technology, State Key Laboratory of Photovoltaic Materials and Cells, Tianjin Key Laboratory of Efficient Solar Energy Utilization, Ministry of Education Engineering Research Center of Thin Film Photoelectronic Technology, Nankai University, Tianjin 300350, China; Frontiers Science Center for New Organic Matter, Nankai University, Tianjin 300071, China; Academy for Advanced Interdisciplinary Studies, Nankai University, Tianjin 300071, China

**Keywords:** photoelectrochemical CO_2_ reduction, Cu_2_O photocathode, TiO_2_, selectivity

## Abstract

Photoelectrochemical carbon dioxide reduction (PEC CO_2_R) enables direct solar-to-chemical conversion. However, few photocathodes are intrinsically stable light absorbers. The application of protective layers remains a critical approach for stabilizing photocathodes in corrosive environments. TiO_2_ is the most widely adopted stabilizer, yet its influence on catalytic performance remains poorly understood. Here, we examine TiO_2_-protected Cu_2_O photocathodes integrated with Au, Cu and Bi cocatalysts, combining experiments and simulations to unravel the role of the TiO_2_ overlayer. We find that TiO_2_ profoundly alters catalytic selectivity, suppressing CO and multicarbon (C_2_^+^) pathways, while its impact on formate production with Bi, In and Sn cocatalysts is comparatively negligible. These results demonstrate that TiO_2_ is not an inert stabilizer, but an active component that reshapes interfacial reaction pathways. This work establishes critical design principles for integrating protective layers with photocathodes to achieve selective and efficient PEC CO_2_R.

## Teaser

This study reveals that the TiO_2_ protection layer is an active component that reshapes interfacial reaction pathways in photoelectrochemical CO_2_ reduction, rather than an inert stabilizer.

## INTRODUCTION

The massive consumption of fossil fuels in human activities has led to a notable increase in the concentration of greenhouse gases, primarily carbon dioxide (CO_2_), in the atmosphere. This surge has contributed significantly to global climate change, such as rising temperatures [1]. In response to this pressing issue, there has been a growing emphasis on implementing strategies to diminish fossil-fuel dependency and mitigate greenhouse gas emissions. One promising approach is implementing CO_2_-reduction technologies, which not only aid in mitigating environmental impacts, but also facilitate the conversion of CO_2_ into valuable products. Among various CO_2_-reduction strategies, photoelectrochemical carbon dioxide reduction (PEC CO_2_R) stands out for its direct utilization of sunlight as an energy source and water as a proton source for CO_2_R [2]. This approach minimizes the losses typically incurred during energy-conversion and energy-transmission processes [3]. Furthermore, the separation of the cathode and anode components in PEC systems helps prevent the undesirable mixing of oxidation and reduction products [4]. An additional advantage of PEC systems lies in the electric field they generate, which facilitates the directional migration of electrons and holes. This phenomenon enhances the carrier separation efficiency within the system [5]. Moreover, the integration of catalysts and light-absorbing materials onto conductive substrates in PEC photoelectrodes renders them amenable to amplification and scalability, further enhancing their utility. Thus, PEC CO_2_R has emerged as a promising avenue for addressing carbon dioxide conversion and solar energy storage, holding significant potential for sustainable energy solutions and environmental remediation efforts [6].

The photocathode is the core component in the PEC CO_2_R system [7]. It needs to have the dual responsibility of efficiently absorbing solar energy to generate a large number of photoelectrons, while concurrently catalysing CO_2_R toward target products [8,9]. Numerous photocathode materials such as Si [10], Sb_2_Se_3_ [11], Cu_2_O [12] and chalcogenide [13] materials have been used in the design of photocathodes. However, a notable challenge arises, as these light-absorbing materials are often unable to maintain stability during the PEC process, primarily due to PEC corrosion in the solution with bias [14,15]. To address this vulnerability, a common strategy involves incorporating a protective layer of titanium dioxide (TiO_2_) onto the outer surface of the photocathode [10,14]. The dense TiO_2_ protective layer plays a dual role: safeguarding the photocathodes and facilitating the extraction of photogenerated electrons.

While TiO_2_ has proven effective as a protective layer for photocathodes in PEC water splitting [16,17], its role in PEC CO_2_R is markedly different [18]. Despite the availability of efficient photoelectrodes designed for PEC water splitting, simply replacing the hydrogen evolution reaction (HER) cocatalyst with one favoring CO_2_R does not yield the anticipated performance. The disparity arises from the fact that CO_2_R kinetics are generally slower, necessitating a larger overpotential compared with the HER. To overcome this hurdle, the use of efficient and selective cocatalysts has become imperative to enhance the activity of CO_2_R by lowering its energy barrier. However, even with advanced CO_2_R cocatalysts, the HER often remains dominant [19,20]. For instance, Ager *et al.* reported H_2_ as the primary product from p-Si/TiO_2_/Cu photocathodes [19]. While an fluorine-doped tin oxide (FTO)/Au/Sb_2_Se_3_/TiO_2_/Au photocathode achieved a CO Faradaic efficiency (FE) of 30% at −0.57 vs. a reversible hydrogen electrode (RHE), H_2_ was still a major co‑product [20]. This challenge is further illustrated by the case of Sn/SnO*_x_* deposited on a Cu_2_O photoelectrode, which generates CO and formate, but also produces a substantial amount of H_2_, with a FE of >40% [21]. This underscores that efficient PEC CO_2_R requires more than a simple combination of a photoelectrode with satisfactory photoelectric conversion performance and a selective electrochemical CO_2_R cocatalyst. Instead, the key lies in a synergistic co‑design of the photoelectrode and the cocatalyst to optimize their interfacial interplay for successful PEC CO_2_R outcomes.

In this study, we delved into the crucial role of a TiO_2_ protective layer on photocathodes for PEC CO_2_R. We chose TiO_2_-protected Cu_2_O photocathodes as representative photoelectrodes and explored three distinctive electrocatalysts—Au, Cu and Bi—corresponding to CO, multicarbon products and formate, respectively [22]. The experimental framework involved depositing Au, Cu and Bi cocatalysts onto TiO_2_-protected Cu_2_O photocathodes and examining the resultant selectivity in PEC CO_2_R. To comprehensively assess the impact of the TiO_2_ layer beneath the cocatalyst on CO_2_R selectivity, we further conducted electrocatalytic experiments. To gain deeper insights, we additionally employed theoretical calculations to elucidate how TiO_2_ alters the original selectivity of a metal cocatalyst by influencing the adsorption energy of key reaction intermediates.

Integrating experimental observations with theoretical simulations, we identified that the selectivity of the photoelectrode is intricately determined by the combined influence of the TiO_2_ protective layer and the cocatalyst. Specifically, when the cocatalyst is deposited on the TiO_2_ protective layer, the interaction between the two components induces changes in the adsorption energy of key intermediates compared with the catalyst itself for these intermediates. This interaction significantly influences the selectivity during the PEC CO_2_R process. Notably, TiO_2_ profoundly alters the catalytic selectivity of Au and Cu, suppressing the CO and multicarbon (C_2_^+^) pathways, respectively, while its impact on formate production with Bi is comparatively negligible. We further expanded our investigation by measuring the PEC CO_2_R performance of In and Sn (elements akin to bismuth)-decorated Cu_2_O photocathodes with a TiO_2_ protection layer, which also exhibit high selectivity for formate production. The Cu_2_O photocathode with a Sn cocatalyst has effectively suppressed the FE of H_2_ to 7.1% at 0 V vs. RHE. Our findings contribute valuable knowledge for the rational design of photoelectrodes for PEC CO_2_R, emphasizing the critical interplay between the TiO_2_ protective layer and cocatalysts in determining the selectivity of the PEC CO_2_R process.

## RESULTS AND DISCUSSION

**Figure 1.**
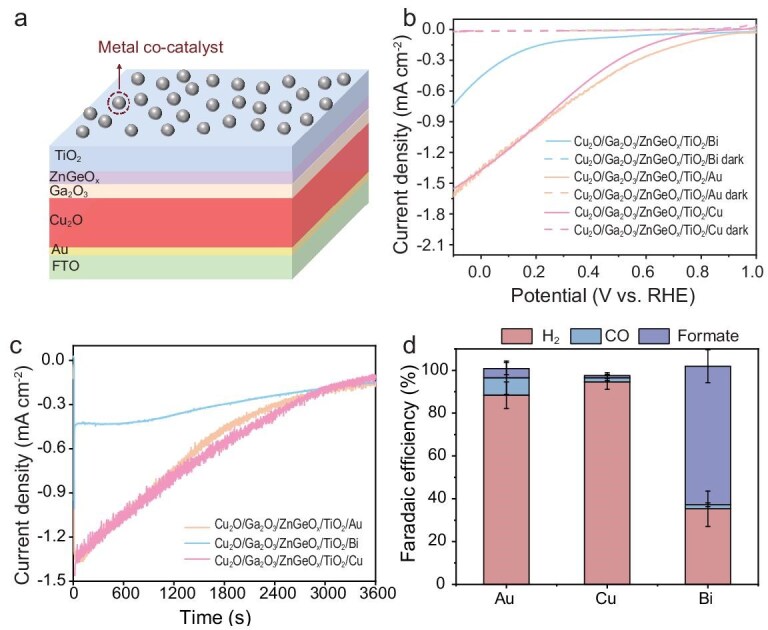
PEC performance of Cu_2_O/Ga_2_O_3_/ZnGeO*_x_*/TiO_2_ photocathodes with Au/Cu/Bi as cocatalyst. (a) Illustration of the structure of the Cu_2_O/Ga_2_O_3_/ZnGeO*_x_*/TiO_2_/metal cocatalyst photocathode. (b) Linear sweep voltammetry of Cu_2_O/Ga_2_O_3_/ZnGeO*_x_*/TiO_2_ photocathodes with Au/Cu/Bi as cocatalyst. (c) Chronoamperogram of the Cu_2_O/Ga_2_O_3_/ZnGeO*_x_*/TiO_2_ photocathode with Au/Cu/Bi as cocatalyst at 0 V vs. RHE. (d) Faradaic efficiency of formate, carbon monoxide and hydrogen on the optimized Cu_2_O/Ga_2_O_3_/ZnGeO*_x_*/TiO_2_ photocathodes with Au/Cu/Bi as cocatalyst at 0 V vs. RHE.

The TiO_2_-protected Cu_2_O photoelectrodes were prepared according to our previous report [23]. Au, Cu and Bi metals, known as distinctive catalysts generating CO, multicarbon products, and formate during electrochemical CO_2_R [22], were employed as cocatalysts on TiO_2_-protected Cu_2_O photoelectrodes by using a thermal evaporation technique. The final structure of the Cu_2_O photocathodes (Cu_2_O/Ga_2_O_3_/ZnGeO*_x_*/TiO_2_/cocatalyst) is illustrated in Fig. 1a and Fig. S1. Scanning electron microscopy and energy dispersive X-ray spectroscopy (SEM–EDX) mapping showed that the TiO_2_-protected Cu_2_O photocathodes were uniformly coated with Au/Cu/Bi nanoparticles (Fig. S2). X-ray photoelectron spectra (XPS) were further carried out to characterize the chemical states of the elements, as shown in Figs S3–S5. Cocatalyst Cu and Bi are easily oxidized in the air. The Cu 2p and Cu LMM spectra of the Cu_2_O photoelectrodes with Cu exhibited Cu^0^ and Cu^1+^. The Bi 4f spectrum of the Cu_2_O photoelectrodes with Bi demonstrates the existence of metallic Bi^0^ and Bi^3+^. Two peaks in the Au 4f spectra correspond to Au 4f_5/2_ and Au 4f_7/2_ peaks.

The PEC CO_2_R of TiO_2_-protected Cu_2_O photocathodes with various cocatalysts (Cu_2_O/Ga_2_O_3_/ZnGeO*_x_*/TiO_2_ with Au, Cu or Bi) was performed in CO_2_-saturated 0.1 M KHCO_3_ electrolyte (pH 6.8) under AM1.5G (100 mW cm^−2^) illumination in a three-electrode system. The cocatalysts with varying thickness were investigated systematically, as shown in Fig. S6. The linear sweep voltammetry (LSV) curves of the Cu_2_O photocathodes with 10-nm cocatalysts and without cocatalysts are shown in Fig. 1b and Fig. S8a. The photocathode without a cocatalyst exhibited an onset potential of 0.1 V vs. RHE, which is much later than the photocathodes with cocatalysts. The Au- and Cu-coated photoelectrodes showed a positive onset potential at around +0.7 V (vs. RHE). However, the Cu_2_O/Ga_2_O_3_/ZnGeO*_x_*/TiO_2_/Bi photocathode exhibited an onset potential of approximately +0.3 V (vs. RHE). This 0.4-V potential difference highlights the distinct energy barriers associated with the photoelectrochemical reactions facilitated by the different cocatalysts. Figure 1c illustrates the chronoamperometric responses at 0 V vs. RHE for the three photocathodes, revealing a gradual photocurrent decrease over time. The changes in the Cu_2_O photocathodes coated with Cu, Au and Bi cocatalysts after the stability test were characterized by using SEM and XPS measurement, as shown in Figs S2–S5. After the stability test, there were still catalysts on the Cu_2_O photoelectrodes, though they were aggregated. All the signals in the XPS spectra decreased after the stability test, resulting from the loss of the catalyst. The Au 4f spectra remain the same as before the reaction (Fig. S4). The Bi 4f spectra showed more Bi^3+^ compared with before the reaction (Fig. S5), which may relate to the bismuth oxycarbonate layer on the surface under the PEC CO_2_R conditions in the potassium bicarbonate electrolyte [24]. The Ti 2p spectra revealed that the oxidation state of the TiO_2_ did not change, remaining in the Ti^4+^ state, as shown in Figs S3 and S4.

The product distribution of the photocathodes, tested at a potential of 0 V vs. RHE, is depicted in Fig. 1d and Figs S6, S7 and S8c. The main product of the photocathode without a cocatalyst was hydrogen. When the Au and Cu were deposited as cocatalysts on the TiO_2_-protected Cu_2_O photoelectrodes, they predominantly produced H_2_, with minimal CO and formate generation, which is a significant deviation from the electrocatalytic product selectivity of Au and Cu. We further conducted a PEC chronoamperogram test at various applied potentials, as shown in Fig. S9. The product distribution exhibited a similar selectivity across the potential range from −0.1 to 0.2 V (vs. RHE). The effect of the TiO_2_ thickness was further investigated. As shown in Figs S10 and S11, compared with 20 nm of TiO_2_, the 40-nm TiO_2_ layer improved the stability of the Cu_2_O/Ga_2_O_3_/ZnGeO*_x_*/TiO_2_ photocathode with the Cu and Au cocatalyst, while maintaining nearly unchanged selectivity, with H_2_ as the dominant product. In contrast, when coated with only 5 nm of TiO_2_, the photocathode suffered from corrosion and the total FE failed to reach 100%. However, for the Bi-coated TiO_2_-protected photoelectrode, formate was the predominant product, aligning with the behavior of Bi in the electrocatalysis. The product distribution suggests that the selectivity of the Cu_2_O/Ga_2_O_3_/ZnGeO*_x_*/TiO_2_/Au and Cu_2_O/Ga_2_O_3_/ZnGeO*_x_*/TiO_2_/Cu photocathodes is influenced not only by the intrinsic selectivity of the catalyst, but also by the interaction of the cocatalyst and the photoelectrode, leading to a relatively low overpotential for the HER and a more positive onset voltage. This suggests that, for some catalysts, when used as cocatalysts deposited on a TiO_2_-protected photoelectrode, their selectivity is no longer determined solely by themselves, but by the cocatalyst and the TiO_2_ protective layer together. In this case, designing a photoelectrode for CO_2_R requires a holistic approach that considers both the cocatalyst and the outer surface of the photoelectrode. Simply combining an efficient photocathode with a catalyst capable of reducing CO_2_ to the desired product is not sufficient. The Cu_2_O photoelectrode is a buried-junction-structure photoelectrode with TiO_2_ serving as an outer protective layer which is not completely covered by the cocatalyst. This suggests that the TiO_2_ layer likely influences the inherent selectivity of the cocatalyst and the combined effect of the TiO_2_ protective layer and the cocatalyst determines the distribution of the products.

To check the influence of different light-absorbing layers, we also performed PEC CO_2_R tests using TiO_2_-coated silicon photocathodes with deposited Au/Cu/Bi as cocatalysts, and their performance is depicted in Fig. S12. Schematic diagrams illustrating the energy-band structure of the Si photocathodes, as presented in prior research [25], are depicted in Fig. S13. The product distribution reveals a consistent trend with the experiments on TiO_2_-protected Cu_2_O photocathodes: a noticeable increase in the proportion of hydrogen gas in the product distribution compared with their electrocatalytic counterparts. The similar trends in product distribution observed across various photocathode materials indicate that the TiO_2_ protective layer and the cocatalyst play a crucial role in the selectivity of the PEC CO_2_R process, regardless of the type of semiconductor photo-absorber material used in the photocathode.

**Figure 2.**
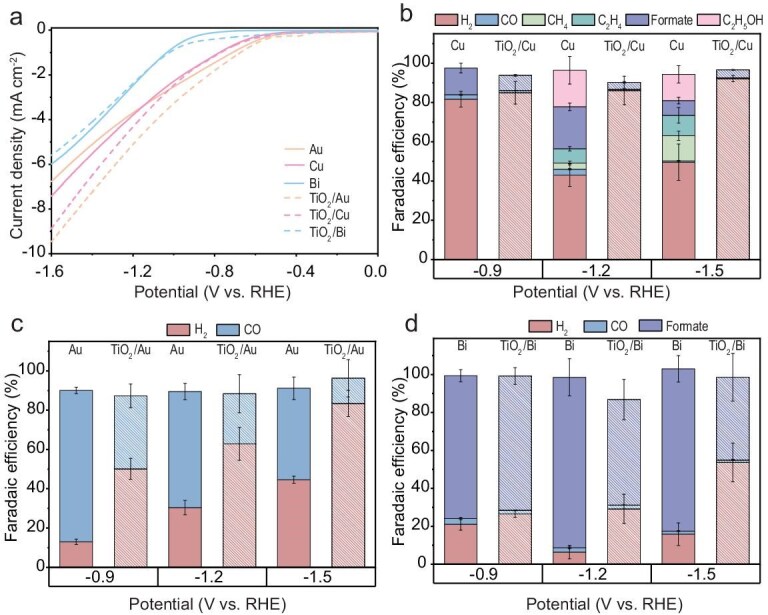
EC performance of Cu/Au/Bi deposited on GC and GC–TiO_2_. (a) LSV curves of GC and GC-TiO_2_ electrodes with Cu/Au/Bi as cocatalysts. (b–d) Faradaic efficiency of GC and GC–TiO_2_ electrodes with (b) Cu, (c) Au and (d) Bi as cocatalysts.

To verify the role of TiO_2_ in altering the selectivity of cocatalysts, three metal catalysts (Cu, Au and Bi) were deposited onto glassy carbon (GC) conductive substrates, both with and without a TiO_2_ layer, for EC CO_2_R. The performance of the bare GC and GC–TiO_2_ is illustrated in Fig. S14. Regardless of whether the bare GC electrode or the GC–TiO_2_ electrode was used, H_2_ was the primary product, with only trace amounts of CO and formate produced. The LSV curves of the six different samples are displayed in Fig. 2a. No significant difference was observed in the onset potential or current density between any of the three metal catalysts, whether TiO_2_ was present or not. Figure 2b–d illustrates the comparative product selectivity of the catalysts Cu, Au and Bi across various potentials, in both the presence and absence of a TiO_2_ layer. The results revealed notable differences in the HER for Au and Cu catalysts when deposited on TiO_2_-coated electrodes. The generation of H_2_ was significantly enhanced at different potentials compared with the catalysts deposited on GC electrodes. In contrast, for the Bi catalyst, although the presence of TiO_2_ led to a slightly increased HER, the main product remained as formate. These results are consistent with the selectivity results from PEC-CO_2_R shown in Fig. 1, indicating that the TiO_2_ layer in the photoelectrode plays a crucial role in interacting with the cocatalysts and affecting the selectivity of the products.

**Figure 3.**
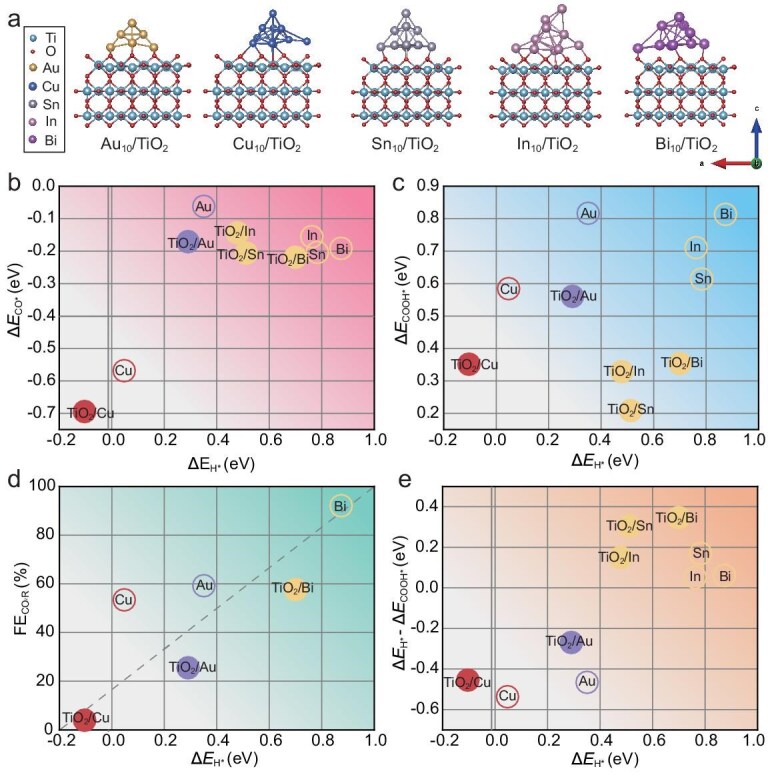
Theoretical calculation of adsorption energy of intermediates with different catalysts. The catalysts combined with TiO_2_ are marked as solid circles of the corresponding color. (a) Optimized metal cluster–TiO_2_ model, depicted from left to right, comprises Au_10_/TiO_2_, Cu_10_/TiO_2_, Sn_10_/TiO_2_, In_10_/TiO_2_ and Bi_10_/TiO_2_. (b) Adsorption energies of the intermediates CO^∗^ and H^∗^. (c) Adsorption energies of the intermediates COOH^∗^ and H^∗^. (d) EC CO_2_R Faradaic efficiency at −1.2 V as a function of Δ*E*_H∗_. (e) Difference between the adsorption energies of H^∗^ and COOH^∗^ as a function of Δ*E*_H∗_.

Previous studies have shown that the adsorption energies of key reaction intermediates involved in CO_2_R tests, such as H^∗^, CO^∗^ and COOH^∗^, can be used to categorize different types of catalysts that lead to different major products in CO_2_R [26]. To elucidate the mechanisms behind the altered selectivity of catalysts in the presence of TiO_2_, the adsorption energies of several key intermediates were calculated through density functional theory (DFT) simulations for representative metal catalysts of different types, in both the presence and absence of TiO_2_. Given that the most stable crystal surface for the five metals is the (111) facet, we used this surface as the catalytic reference. The model of metals supported by TiO_2_ was constructed by a metal cluster (10) sitting on a (4 × 2) TiO_2_ (110) surface exposed at the oxygen end and the bottom of the two layers of the TiO_2_ layers were fixed. The optimized metal cluster–TiO_2_ model comprising Au_10_/TiO_2_, Cu_10_/TiO_2_, Sn_10_/TiO_2_, In_10_/TiO_2_ and Bi_10_/TiO_2_ is depicted in Fig. 3a. In Fig. 3b, it can be observed that the elements mainly produce C1 products that remain in the upper-right region, even after interaction with TiO_2_. However, Cu, being the only metal cocatalyst with a slightly strong adsorption energy for CO^∗^ to achieve C–C coupling, moves to the lower-left region when deposited on TiO_2_, indicating that H_2_ gas will become the primary product. Figure 3c shows that the presence of TiO_2_ increases the adsorption energies of COOH^∗^ and H^∗^ for all the catalysts. This facilitates the adsorption of H^∗^, leading to an increase in H_2_ production when these catalysts are present with TiO_2_. The increase in the COOH^∗^-adsorption energy does not reverse this trend, suggesting that the adsorption energy for H^∗^ more significantly influences the selectivity between the CO_2_R and competitive HER in the presence of TiO_2_. This leads us to investigate the CO_2_R Faradaic efficiency at −1.2 V as a function of Δ*E*_H∗_ (Fig. 3d). It can be observed that the Faradaic efficiency of CO_2_R is approximately linearly positively correlated with *E*_H∗_. Furthermore, we conducted further analysis on the changes and impacts of the difference in adsorption energies between H^∗^ and COOH^∗^ in the presence of TiO_2_, as shown in Fig. 3e. Cu, Au and TiO_2_/Au all have negative differences, indicating that they inevitably produce H_2_ gas while undergoing CO_2_R. On the other hand, Bi, In and Sn, which primarily yield formic acid, have positive differences in the presence or absence of TiO_2_. This suggests that, even though the interaction between TiO_2_ and these metals makes H^∗^ adsorption easier, they still have the potential to undergo CO_2_R with close to 100% Faradaic efficiency. To further elucidate the electronic-structure origin of the TiO_2_-induced selectivity modulation, we performed interfacial charge-transfer and projected density-of-states analyses, as shown in Fig. S15. The TiO_2_ protective layer tunes selectivity through an element-dependent electronic modulation mechanism: it induces a d-band upshift in Cu to trigger the HER by strengthening the H-binding, while inducing a p-band downshift in In to optimize the COOH^∗^ interactions, thereby preserving high formate selectivity. Therefore, the influence of TiO_2_ on the CO_2_R selectivity of different catalysts can be described as follows: (i) the interaction between the TiO_2_ and the cocatalysts results in selectivity altering for the PEC CO_2_R compared with their individual electrocatalytic performance; (ii) when the catalyst is deposited on TiO_2_, its adsorption energy for H^∗^ moves towards the direction more suitable for the HER, resulting in enhanced H_2_ production; (iii) when deposited on TiO_2_, Cu predominantly generates H_2_ due to its suitable H^∗^-adsorption energy for the HER, while the H_2_ evolution of Au is enhanced due to its favorable H^∗^ adsorption. Bi, despite its negative-shifted H^∗^-adsorption energy, remains in a region where H^∗^ adsorption is less favorable and its stronger COOH^∗^ adsorption leads to formic acid as the primary product.

**Figure 4.**
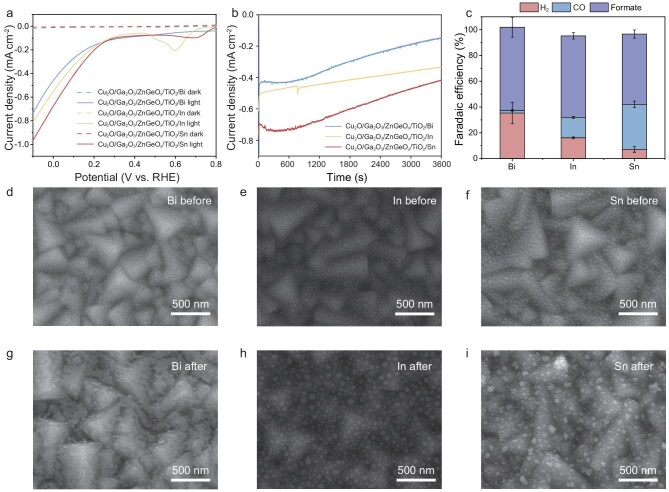
PEC performance and morphological characterizations of Cu_2_O/Ga_2_O_3_/ZnGeO*_x_*/TiO_2_ photocathode with Bi/In/Sn as cocatalyst. (a) LSV of Cu_2_O/Ga_2_O_3_/ZnGeO*_x_*/TiO_2_ photocathode with Bi/In/Sn as cocatalyst. (b) Chronoamperogram of Cu_2_O/Ga_2_O_3_/ZnGeO*_x_*/TiO_2_ photocathode with Bi/In/Sn as cocatalyst at 0 V vs. RHE. (c) Faradaic efficiency of Cu_2_O/Ga_2_O_3_/ZnGeO*_x_*/TiO_2_ photocathode with Bi/In/Sn as cocatalyst at 0 V vs. RHE. (d) SEM image of the surface of Cu_2_O/Ga_2_O_3_/ZnGeO*_x_*/TiO_2_/Bi photocathode (d) before and (g) after chronoamperogram test, Cu_2_O/Ga_2_O_3_/ZnGeO*_x_*/TiO_2_/In photocathode (e) before and (h) after chronoamperogram test, Cu_2_O/Ga_2_O_3_/ZnGeO*_x_*/TiO_2_/Sn photocathode (f) before and (i) after chronoamperogram test.

When the adsorption energies depicted in Fig. 3 are analysed, it is noticed that In and Sn, similarly to Bi, exhibit weak H^∗^-adsorption energy when combined with TiO_2_, yet they demonstrate a preference for COOH^∗^ adsorption over H^∗^. This suggests that these catalysts still possess the potential to selectively produce formic acid on TiO_2_-protected Cu_2_O photocathodes. To validate this hypothesis, PEC CO_2_R experiments were conducted using Cu_2_O photocathodes with In and Sn as cocatalysts. The performance of the Cu_2_O/Ga_2_O_3_/ZnGeO*_x_*/TiO_2_ photocathodes with Bi/In/Sn for photoelectrochemical CO_2_R was analysed. As illustrated in Fig. 4a, the onset potentials and photocurrents of the electrodes with these cocatalysts were comparable. Figure 4c and Fig. S16 confirm that formate was the predominant product for all three photocathodes. However, the CO-production percentages varied among the cocatalysts, with Bi, In and Sn yielding 2%, 16% and 35%, respectively. This difference indicates that In and Sn can adsorb more H^∗^ than Bi during PEC CO_2_R, which is consistent with their more negative adsorption energy for H^∗^ relative to Bi. Although CO_2_R can be carried out with high selectivity, the photocurrents were much lower than those used for water splitting, due to the slow kinetics of CO_2_R. Additionally, it was observed that, during the chronoamperometry tests, the photocurrent of each photocathode decreased over time and there was a trend towards an increased proportion of gaseous products, particularly H_2_ (Fig. S17). Nevertheless, no dark currents were detected in the LSV curve after testing, indicating that these electrodes were still well covered by TiO_2_ and did not suffer significant corrosion (Fig. S18). The SEM images and XPS spectra presented in Fig. 4d–i and Fig. S19 indicate that the catalysts aggregated, with an increased presence of Sn^0^ and Sn^2+^ after the reaction. In contrast, the oxidation state of the TiO_2_ did not change, remaining as Ti^4+^. Therefore, the reduction in the active sites may account for the decrease in the photocurrent and the increase in the H_2_ evolution [27]. This can be potentially solved by re-catalysing the photoelectrode surface or using cocatalysts that bind more strongly to the TiO_2_ and do not agglomerate. However, during extended 2-hour stability testing, the FE of the Cu_2_O photocathodes with Sn cocatalyst decreased significantly to <100% in the second hour due to corrosion (Fig. S20). The liquid product of the ^13^C-isotope-labeling experiment was analysed by using nuclear magnetic resonance (NMR). As shown in Fig. S21, the peak splitting (*J*_C–H_ coupling) in the ^1^H NMR spectra demonstrate that the product, formate, is derived from CO_2_. Increasing the thickness of the TiO_2_ to 40 nm in the Cu_2_O/Ga_2_O_3_/ZnGeO*_x_*/TiO_2_/Sn photocathodes resulted in a slight increase in the FE for H_2_, while the selectivity toward CO_2_R remained high, as shown in Fig. S22. Conversely, a 5-nm TiO_2_ coating, similar to the results observed in Cu_2_O photocathodes with Cu or Au cocatalysts, led to corrosion of the photocathode. We further conducted a systematic investigation of the performance of the Cu_2_O/Ga_2_O_3_/ZnGeO*_x_*/TiO_2_/Sn photocathodes at different potentials, as shown in Fig. S23. The optimal FE for formate production was obtained at 0 V (vs. RHE). Notably, the total FE failed to reach 100% at −0.1 V (vs. RHE), potentially due to electrode corrosion under these conditions. Compared with previously reported studies, the Cu_2_O photocathodes with Bi/In/Sn demonstrated an enhanced FE of formate for CO_2_RR at a more positive potential, as indicated in Table S3.

## CONCLUSION

In conclusion, we uncovered that the selectivity of the TiO_2_-protected photoelectrodes for PEC CO_2_R is determined by both the TiO_2_ protective layer and the cocatalyst. The interaction between the cocatalyst and TiO_2_ will change the adsorption energy of key intermediates, thus affecting the selectivity. Notably, the influence on formate generation, particularly with cocatalysts such as Bi, In and Sn, is minimal, with formate remaining the main product. In contrast, cocatalysts such as Cu and Au, known for their role in producing multicarbon and CO products, exhibit an enhancement in the HER, attributable to the increased adsorption energy of H^∗^. This emphasizes that, in order to design an efficient CO_2_R photoelectrode, it is crucial to rationally design the cocatalyst to obtain the appropriate adsorption energy for key intermediates. Additionally, future efforts should focus on replacing or coating the TiO_2_ layer with alternative stable protective materials that do not compromise PEC CO_2_R performance, such as conductive polymers or the recently reported Ta_2_O_5_ [19]. Applying a thick catalyst layer to fully cover the TiO_2_ along with a back-illuminated structure may also help to suppress the HER [28]. Furthermore, DFT modeling provides a powerful tool for screening other potential protection layers suitable for CO_2_R. Our findings contribute new insights to the knowledge base, offering valuable guidance for the design of highly efficient and selective photoelectrodes with protection layers for PEC CO_2_R applications.

## Supplementary Material

nwag092_Supplemental_File
