# Supplementary material for: TiO2 protective layers shape photocathode performance in photoelectrochemical CO2 reduction
Source: Natl Sci Rev. 2026 Feb 9;13(8):nwag092. doi: 10.1093/nsr/nwag092 (PMC13142158; doi:10.1093/nsr/nwag092)
Supplement: nwag092_Supplemental_File [file nwag092_supplemental_file.pdf]

Supporting Information

TiO_2 Protective Layers Shape Photocathode Performance in Photoelectrochemical CO_2 Reduction

Linxiao Wu[1], Yumeng Han[1], Hao Chen[1], Peixuan Liu[2], Jinshui Cheng[1], Bin Shao[2], Jingshan Luo[1,3,4]*

[1]Institute of Photoelectronic Thin Film Devices and Technology, State Key Laboratory of Photovoltaic Materials and Cells, Tianjin Key Laboratory of Efficient Solar Energy Utilization, Ministry of Education Engineering Research Center of Thin Film Photoelectronic Technology, Nankai University, Tianjin 300350, China
[2]College of Electronic Information and Optical Engineering, Tianjin Key Laboratory of Optoelectronic Sensor and Sensing Network Technology, Nankai University, Tianjin 300350, China
[3]Frontiers Science Center for New Organic Matter, Nankai University, Tianjin 300071, China
[4]Academy for Advanced Interdisciplinary Studies, Nankai University, Tianjin 300071, China
*Email: jingshan.luo@nankai.edu.cn

Experimental Section

Chemicals

All the chemicals and reagents were used as purchased without further purification. Chemicals and reagents used in this work are as follows: copper (II) sulfate ($CuSO_4$, ≥99.95%, Sigma-Aldrich), DL-Lactic acid (90%, TCI), potassium phosphate dibasic anhydrous (K_2HPO_4, 99.99%, Aladdin), potassium hydroxide (KOH, 99.999%, Aladdin), potassium bicarbonate ($KHCO_3$, 99.99%, Aladdin), bis(μ-dimethylamino)tetrakis(dimethylamino)digallium (98%, Strem Chemicals), tetrakisdimethylamino titanium (99%, Strem Chemicals), diethylzinc (99.99%, Dongguan NanoFrontier Microelectronic Equipment Co. Ltd), tetramethoxygermane (98%, Alfa Aesar).

Fabrication of Cu_2O photocathodes

20 nm Cr film and 100 nm Au film were successively prepared by DC magnetron sputtering on FTO glass, which was used as the substrate for Cu_2O fabrication. Cr film was served as an adhesive layer. The electroplating solution was a basic solution of lactate stabilized copper sulfate prepared by dissolving 3.192 g $CuSO_4$, 27 g lactic acid and 8.708 g K_2HPO_4 in 100 mL H_2O. Then, the solution pH was adjusted to 12 by a 2 M KOH solution. Cu_2O was electrodeposited with a current density of −0.1mA cm^{-2} for 100 min in a two-electrode system at 30°C. A large piece of Au coated glass was used as the counter electrode. After the electrodeposition, Cu_2O film was washed with plenty of water and dried in N_2 gas.

Ga_2O_3 film, $ZnGeO_x$ film and TiO_2 protective layer were then deposited sequentially onto the Cu_2O film samples with an ALD system (NCE-200R). Bis(μ-dimethylamino)tetrakis(dimethylamino)digallium and water were used as the Ga and oxygen precursor, respectively. The Ga_2O_3 film was deposited by running 200 cycles at 150°C. which gives a film of approximately 20 nm in thickness. The Ga source was heated at 125°C. A 20 nm-thick germanium-doped zinc oxide ($ZnGeO_x$) was deposited by running 40 super cycles consisting of 1 cycle of tetramethoxygermane and water after 3 cycles of diethyl zinc and water, at 120°C. Titaniumdioxide (TiO_2) was deposited at 150°C using tetrakis(dimethylamino)titanium (TDMAT) and H_2O as the Ti and O precursors, respectively. 360 cycles resulted in a thickness of 20 nm. TDMAT was heated to 78°C. The precursor temperatures of Ge, Zn, and O were kept at room temperature.

Co-catalyst deposition and electrolysis sample preparation

A co-catalyst layer composed of Au/Cu/Bi/In/Sn was applied to the Cu_2O photocathodes via vacuum thermal evaporation (PD-400S from PU DI ZHEN KONG). The cocatalyst thickness was determined using a calibrated quartz crystal oscillator. The substrate for the electrolysis was glassy carbon, which was meticulously polished using a crystal orientation grinder and subsequently cleaned with ultrapure water. The TiO_2 coated glassy carbon substrate was prepared by depositing a 20 nm layer of TiO_2 onto the glassy carbon using ALD. Subsequently, 10 nm Au/Cu/Bi was deposited via a same vacuum thermal evaporation method on both the TiO_2 coated and uncoated substrates to fabricate distinct electrolysis samples.

PEC measurement and EC measurement

The PEC measurement was performed in a gas-tight H-cell using an electrochemical workstation (CHI-760E) under AM 1.5 G illumination. The light intensity was calibrated by a silicon diode by adjusting the distance between the light and the cell. Three-electrode system was used including Cu_2O photocathode served as the working electrode, a Pt foil and Ag/AgCl reference used as the counter and reference electrode, respectively. PEC performance was measured in a CO_2-saturated 0.1 M $KHCO_3$ electrolyte with a pH of 6.8. CO_2 was continuously infused into the solution at a constant rate of 10 sccm during the test. EC performance was tested using the same setup as for PEC devices without light illumination. glass carbon (GC)/co-catalyst and GC/TiO_2/co-catalyst were served as the working electrode. A Pt foil and Ag/AgCl reference were used as the counter and reference electrode, respectively.

Product analysis

Gaseous products were quantified every 11 min by online gas chromatograph (790 Plus, FULI INSTRUMENTS). After the stability test, the liquid products were quantified by high-performance liquid chromatography. The calibration curves for product quantification are shown in Figure S24. The electrolyte for ^{13}C isotopic labelling experiments was 0.1 M KOH purged with $^{13}CO_2$. The liquid product formate after 1 h chronoamperogram test was analyzed via ^{1}H NMR by 800 MHz digital NMR

spectrometer (AVANCE NEO 800 MHz, Bruker).

Material characterization

The morphology and elemental analysis were measured by field emission scanning electron microscope (JSM-7800F). X-ray photoelectron spectroscopy (XPS) measurements were carried out with a photoelectron spectrometer (Thermo Scientific ESCALAB 250Xi). All peaks are calibrated using adventitious C 1s peak of 284.8 eV to correct charge shift of binding energies after deconvolution.

Computational detail

The density functional theory method in the Vienna Ab initio Simulation Package (VASP) [1-3] was used to simulate and calculate the adsorption energies. The interaction between electrons and ionic cores and the pseudopotential of the all-electron wave function were described by the projector-augmented wave (PAW) method. The exchange-correlation (XC) potential was treated using the generalized gradient approximation (GGA) in the form of Revised Perdew-Burke-Ernzerhof (RPBE) functional [4]. The van der Waals interactions were described by using DFT-D3 dispersion correction[5]. The adsorption energies were calculated as E_{ad*} - $E_{ad(g)}$ - E_{*}, where E_{ad*}, $E_{ad(g)}$, E_{*} are the total energies of the optimized adsorbate-substrate system, the adsorbate in the gas phase, and the clean substrate. The adsorbates involved in the simulation are H, CO and COOH. For non- gas-phase adsorbates COOH and H, it is necessary to find gas-phase reference molecules as energy references. $E_{COOH(g)}$ was calculated from the gas-phase reference molecules CO_2 and H_2, and $E_{H(g)}$ was calculated from the gas-phase reference molecule H_2 accordingly. The Kohn-Sham equation was solved by using a plane wave basis set which was specified with a cut-off of 500 eV. To ensure the reliability of our chosen cutoff energy, we conducted convergence tests. Table S1 presents the variations in the adsorption energy of COOH at cutoff energies. The results show that the deviation in adsorption energy compared to simulations at higher cutoff energies is within 0.6%. This minimal deviation demonstrates that 500 eV provides accurate results while significantly reducing computation time. The criteria of the force on the atoms in the optimization and of the energy in the self-consistent cycle were less than 0.05 eV/Å and 10^{-5} eV, respectively. The Brillouin zone was sampled using a 2 × 2 × 1 Monkhorst-Pack k-point grid, and the vacuum layer is greater than 10 Å.

To investigate the influence of the TiO_2 substrate on the adsorption of intermediates by metal catalysts, we constructed a metal/TiO_2 model. In this model, the intermediates are initially adsorbed at the same metal site and at the same angle as in the pure metal case. The selected metal site is close to the TiO_2 surface, which allows us to consider the interface effect. From a thermodynamic perspective, the (111) facet is regarded as the most stable surface for Au, Cu, Bi, In, and Sn [6-10]. This facet exhibits the lowest relative energy, making it advantageous for experimental preparation and acquisition. In the context of catalytic processes, the (111) facet is a low-index plane commonly observed in face-centered cubic (fcc) and hexagonal close-packed (hcp) metals. The

atoms on this crystal plane have higher energy and activity, and there are more dangling bonds and unsaturated coordination atoms, especially low-coordinated surface atoms. These characteristics increase the number of surface-active sites, thereby enhancing catalytic activity. Based on these reasons—the thermodynamic stability and catalytic relevance of the (111) facet—we used the (111) facet of the metals as the model for our adsorption studies. For comparative purposes, the metal in the metal/TiO_2 model should ideally exhibit a comparable (111) planar stacking. To achieve this, we considered two modeling methods: plate stacking and cluster stacking, as shown in Figure S25a and Figure S25b.

In Figure S25a, only the metal surface in the c-direction exhibits the (111) planar stacking, similar to the pure metal case. In contrast, each surface of the tetrahedral metal cluster in Figure S25b exhibits a similar (111) facet. Due to the lattice mismatch issues associated with the plate stacking model Figure S25a, we chose the tetrahedral metal cluster model for our calculations. This approach ensures consistency in comparison across five different metal catalysts.

The pure metals were modeled by using a periodic (3 × 3) four-layered fcc (111) slab, and the bottom of the two layers were fixed. Based on these three advantages—thermodynamic stability [11-13], experimental availability [14], and distinctive surface electronic characteristics, we selected the (110) facet of rutile TiO_2 as the substrate for our adsorption model. The model of metals supported by TiO_2 was constructed by a metal cluster(10) sitting on a (4 × 2) TiO_2 (110) surface exposed at the oxygen end, and the bottom of the two layers of the TiO_2 layers were fixed (Figure 3a)，with a coverage of about 10%.[6] Prior to constructing the catalytic model, the adsorption of intermediates (H, CO, and COOH) at various sites—top, bridge, and vacancy—on the pure metal was assessed. The results of these tests are presented in Table S2. The top adsorption site is the most stable for the intermediates on pure metal surfaces. This observation guided our selection of the top adsorption method for simulating intermediate adsorption on pure metal catalysts. The adsorption models of other intermediates on different metals also follow this stability trend for top adsorption. The constructed adsorption model is illustrated in Figure S26.

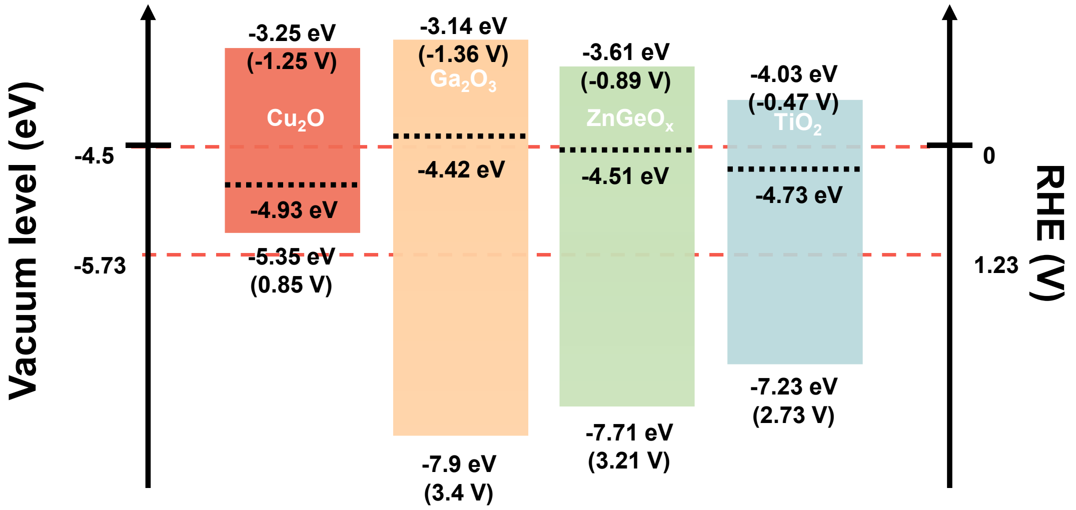

Figure S1. Schematic diagrams of the energy band structure of Cu_2O photocathodes. Reproduced with permission from Cheng et al., Nat Commun 14, 7228 (2023). Copyright 2023 Authors, licensed under a Creative Commons Attribution (CC BY) License.[15]

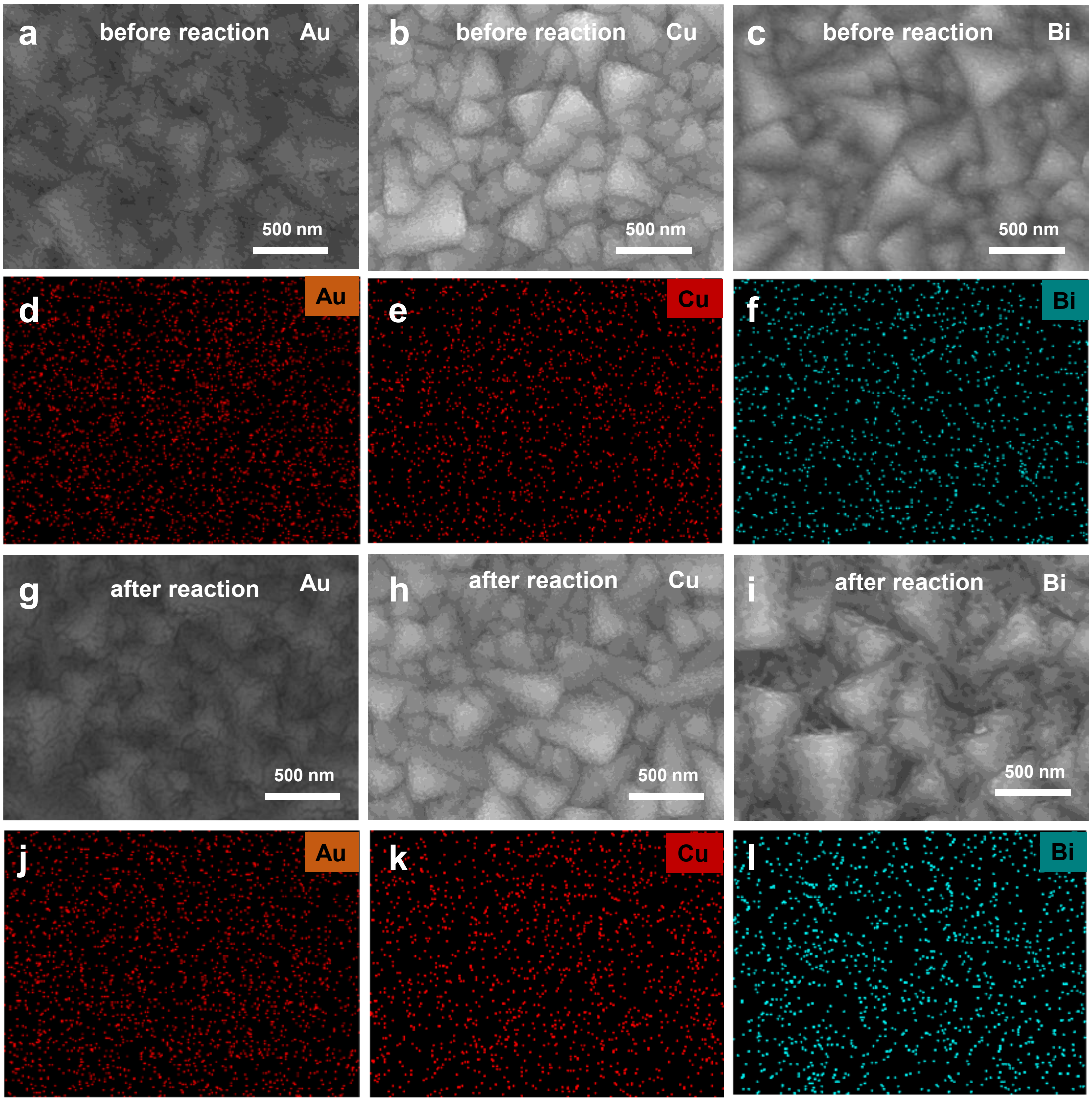

Figure S2. SEM and EDS mapping of Cu_2O photocathodes with Au, Cu and Bi co-catalyst before and after 1h chronoamperogram test.

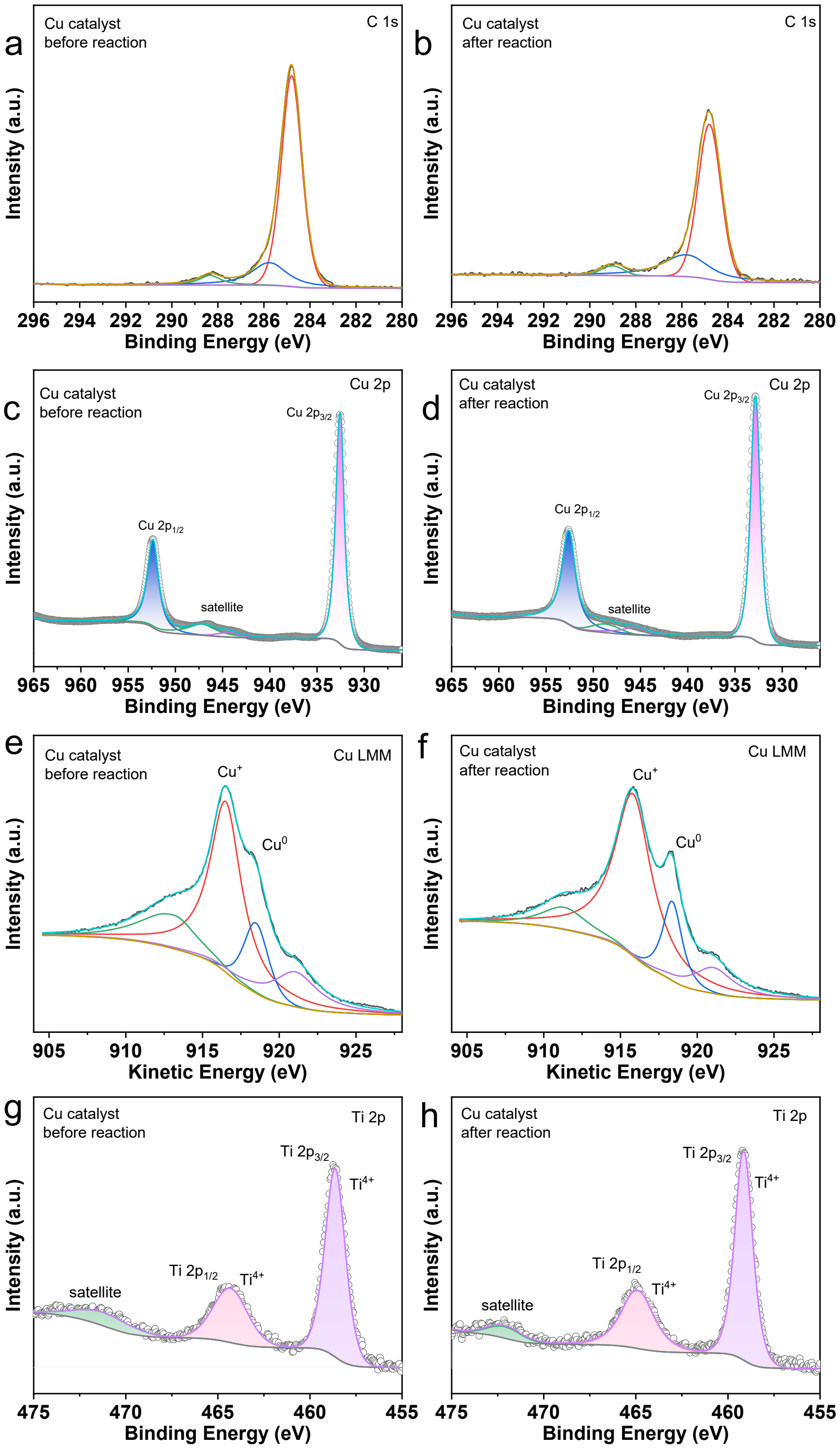

Figure S3. XPS spectra of the Cu_2O photocathodes with Cu as co-catalysts before (a, c, e, g) and after (b, d, f, h) PEC CO_2R test. (a, b) C 1s, (c, d) Cu 2p, (e, f) Cu LMM, (g, h) Ti 2p.

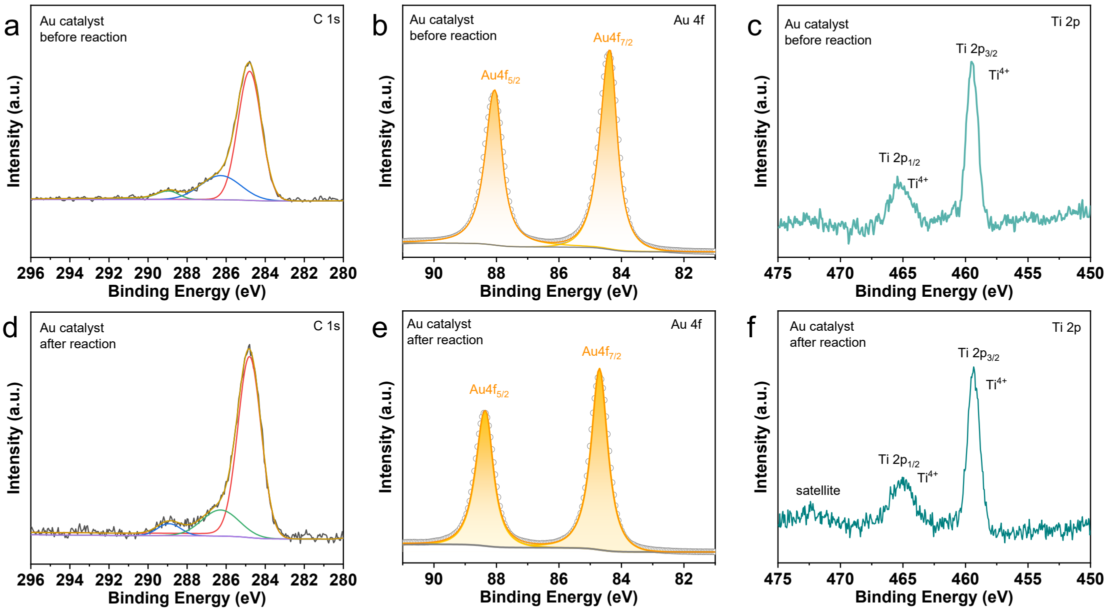

Figure S4. XPS spectra of the Cu_2O photocathodes with Au as co-catalysts before (a, b, c) and after (d, e, f) PEC CO_2R test. (a, d) C 1s, (b, e) Au 4f, (c, f) Ti 2p.

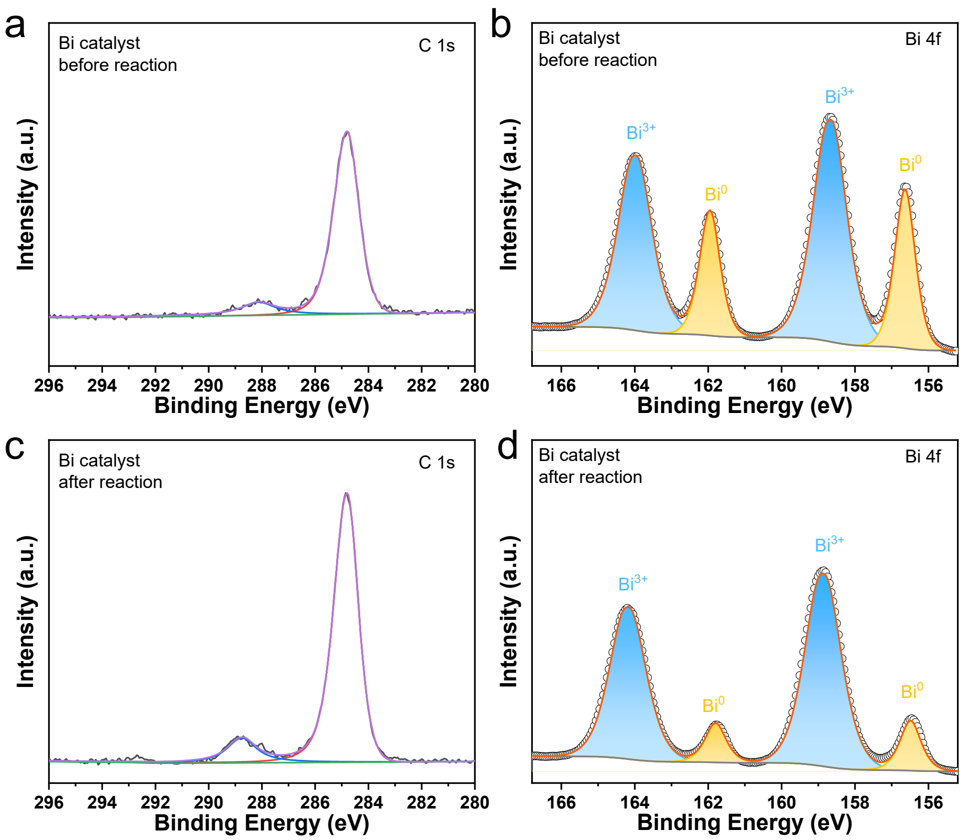

Figure S5. XPS spectra of the Cu_2O photocathodes with Bi as co-catalysts before (a, b) and after (c, d) PEC CO_2R test. (a, c) C 1s, (b, d) Bi 4f.

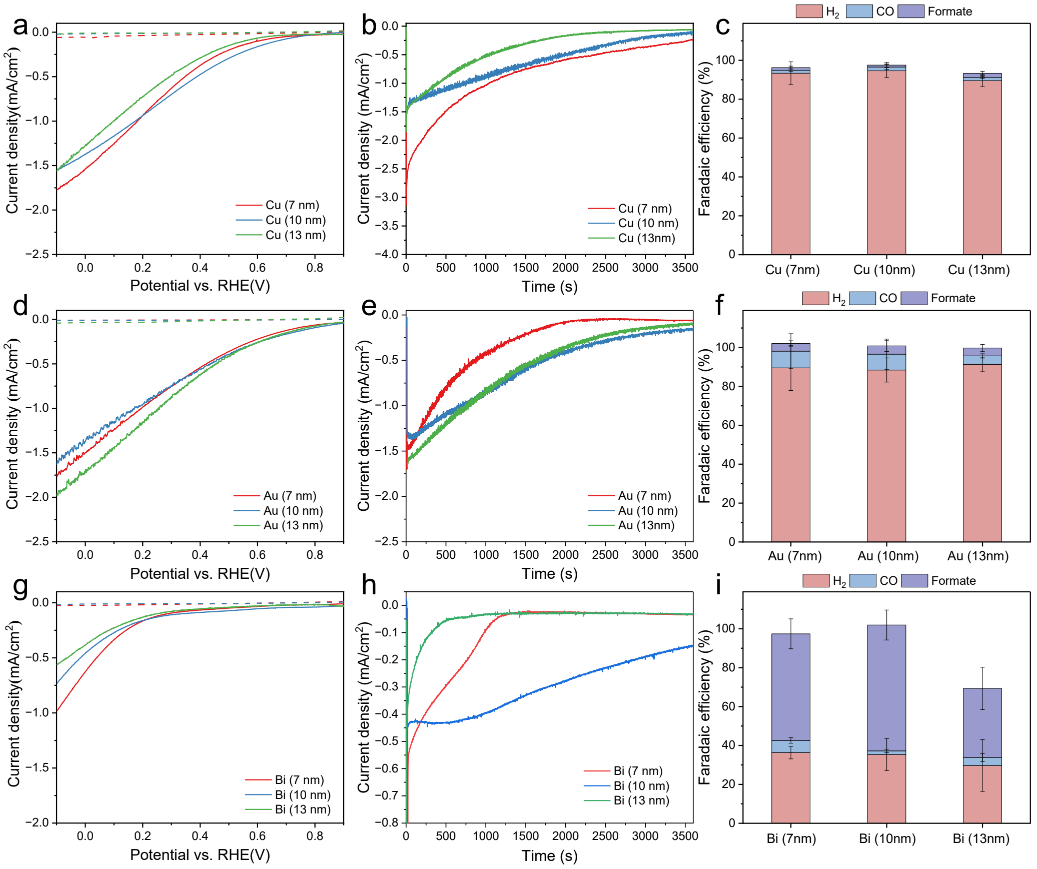

Figure S6. PEC performance of Cu_2O photocathodes with different thickness of Cu (a, b, c), Au (d, e, f), and Bi (g, h, i) cocatalysts. A thicker cocatalyst layer of Bi can block light and reduce the photocurrent, and it can also exacerbate the inherent corrosion, leading to Faradaic efficiencies that fall short of 100%.

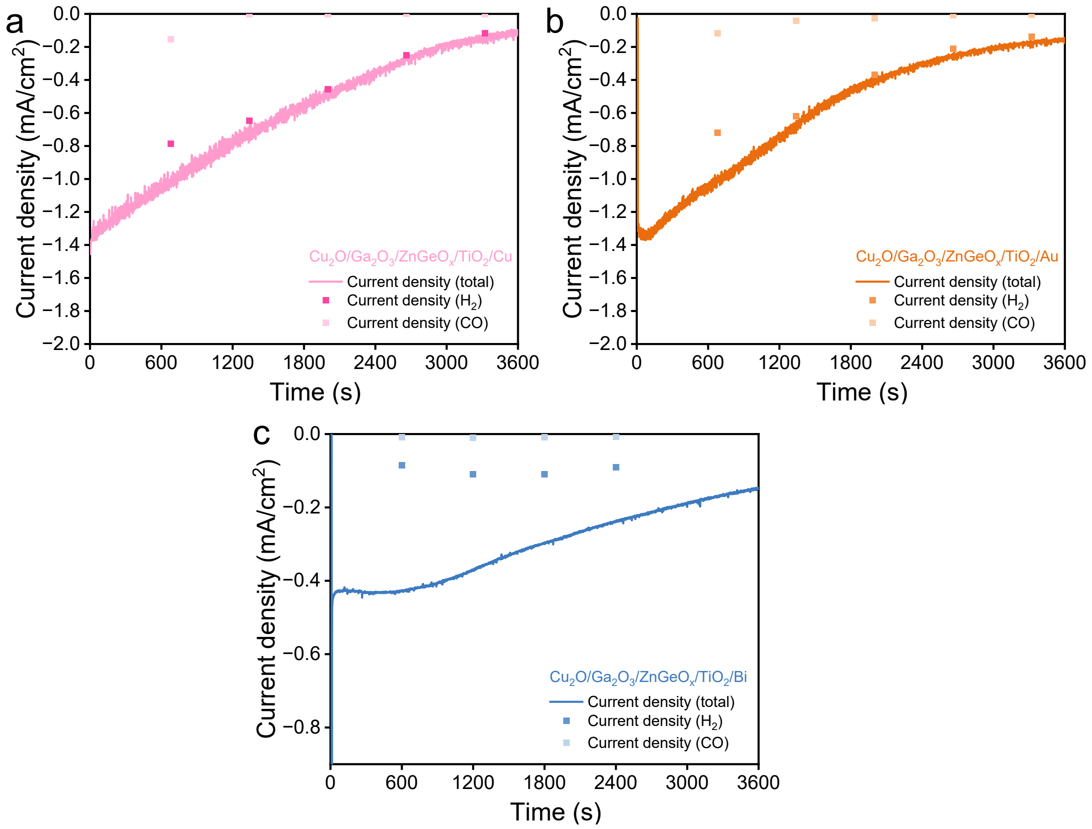

Figure S7. Partial current densities of H_2 and CO of (a) $Cu_2O/Ga_2O_3/ZnGeO_x/TiO_2/Cu$, (b) $Cu_2O/Ga_2O_3/ZnGeO_x/TiO_2/Au$, (c) $Cu_2O/Ga_2O_3/ZnGeO_x/TiO_2/Bi$ at 0 V (versus RHE).

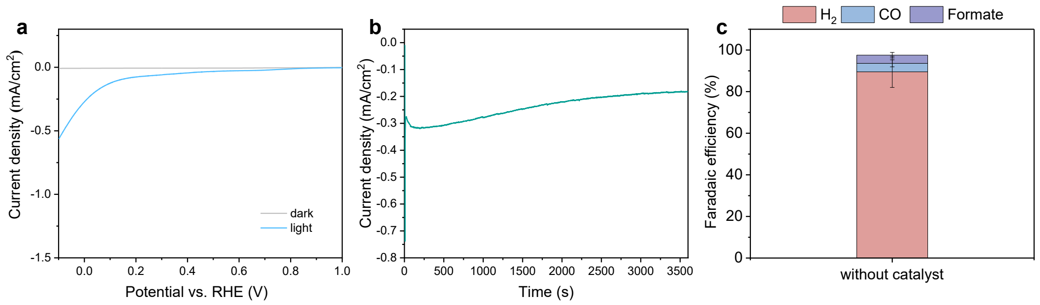

Figure S8. PEC performance of catalyst-free photocathodes.

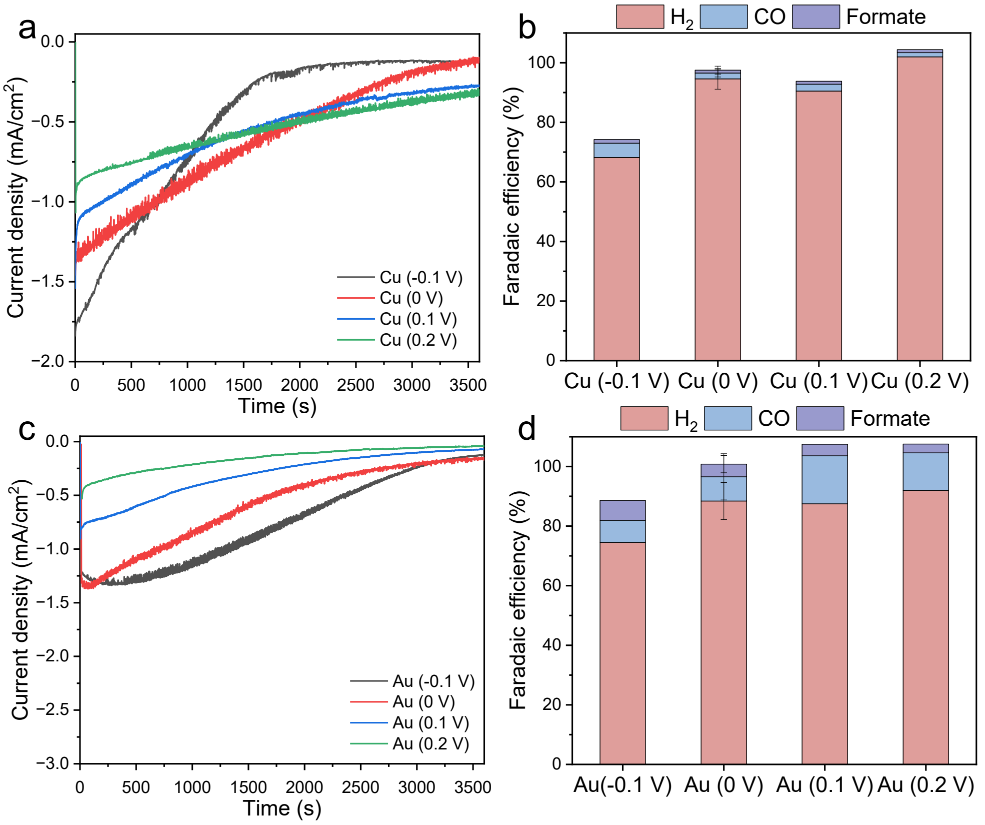

Figure S9. Chronoamperogram test (a) and FE (b) of $Cu_2O/Ga_2O_3/ZnGeO_x/TiO_2/Cu$ photocathodes at different potential under AM 1.5 G illumination. Chronoamperogram test (c) and FE (d) of $Cu_2O/Ga_2O_3/ZnGeO_x/TiO_2/Au$ photocathodes at different potential under AM 1.5 G illumination.

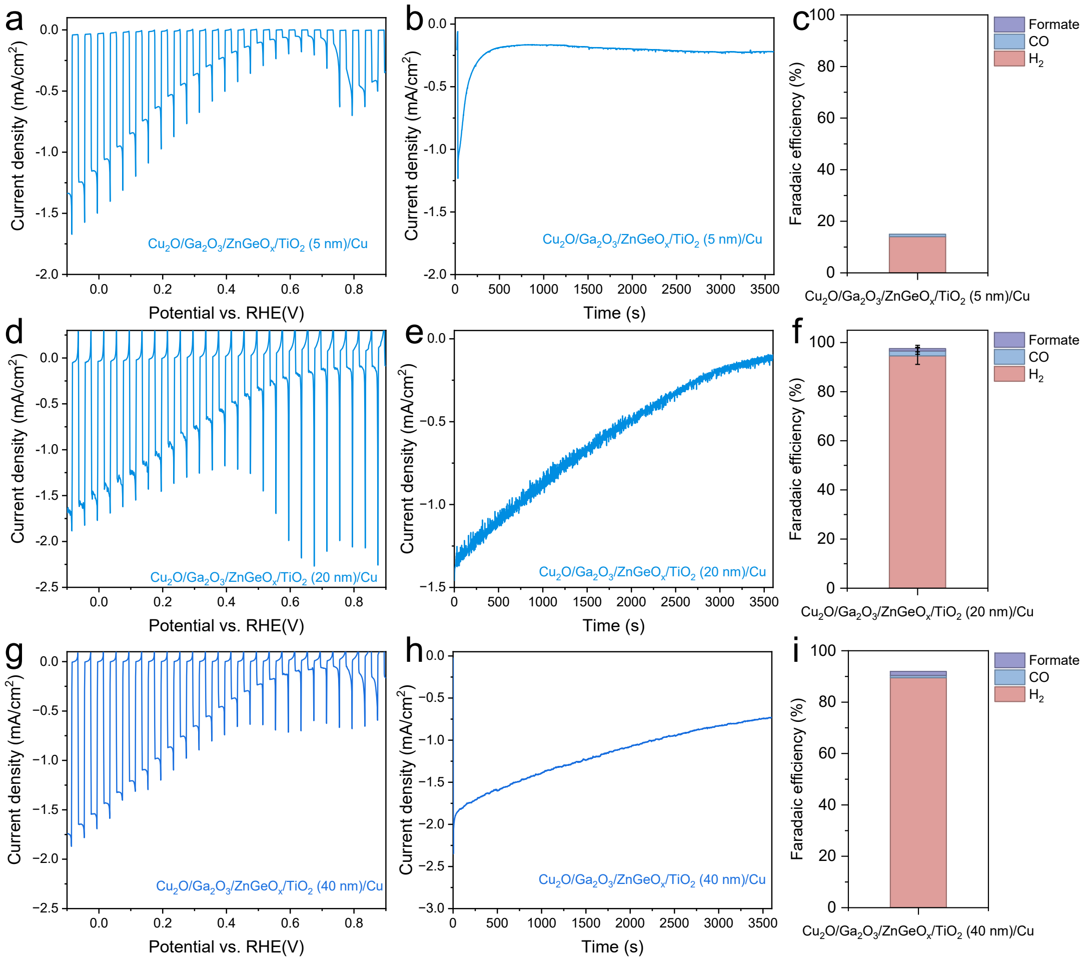

Figure S10. (a) LSV curves, (b) stability test and (c) FE of $Cu_2O/Ga_2O_3/ZnGeO_x/TiO_2$ (5 nm)/Cu photocathodes. (d) LSV curves, (e) stability test and (f) FE of $Cu_2O/Ga_2O_3/ZnGeO_x/TiO_2$ (20 nm)/Cu photocathodes. (g) LSV curves, (h) stability test and (i) FE of $Cu_2O/Ga_2O_3/ZnGeO_x/TiO_2$ (40 nm)/Cu photocathodes.

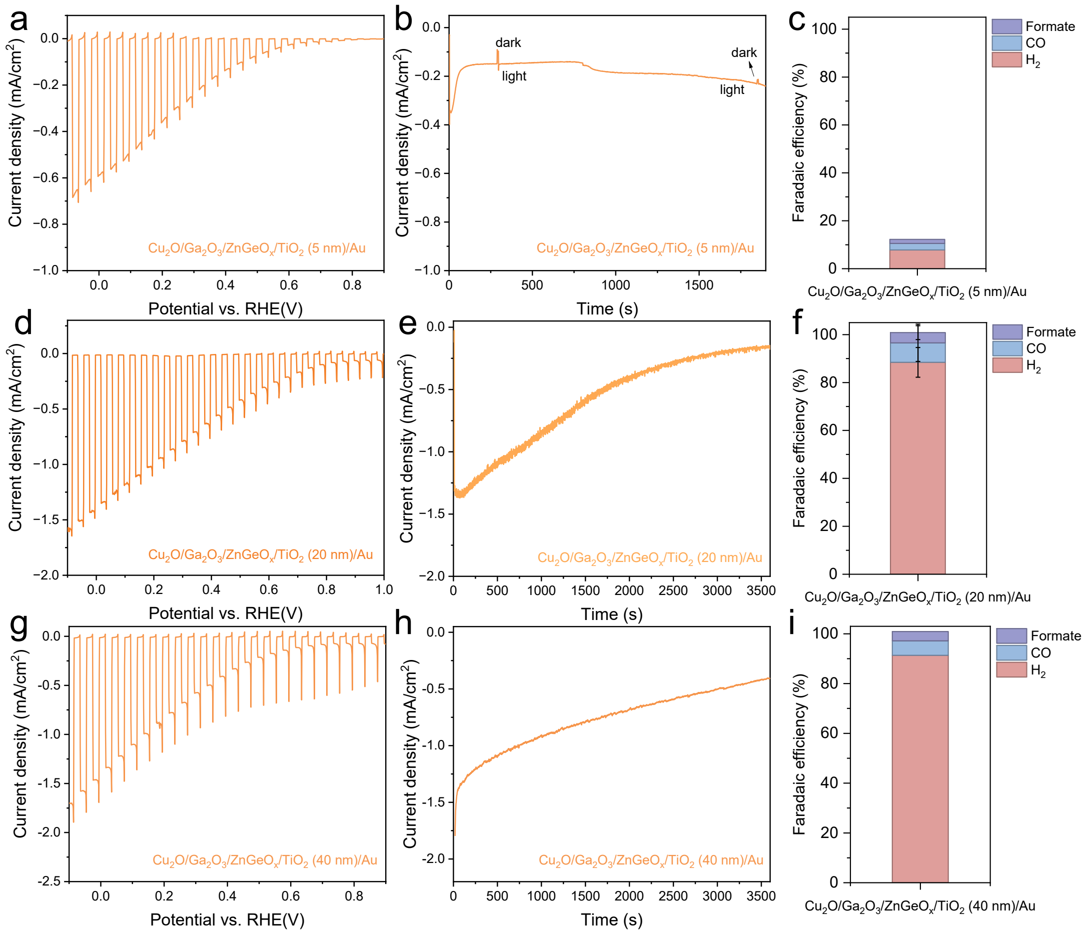

Figure S11. (a) LSV curves, (b) stability test and (c) FE of $Cu_2O/Ga_2O_3/ZnGeO_x/TiO_2$ (5 nm)/Au photocathodes. (d) LSV curves, (e) stability test and (f) FE of $Cu_2O/Ga_2O_3/ZnGeO_x/TiO_2$ (20 nm)/Au photocathodes. (g) LSV curves, (h) stability test and (i) FE of $Cu_2O/Ga_2O_3/ZnGeO_x/TiO_2$ (40 nm)/Au photocathodes.

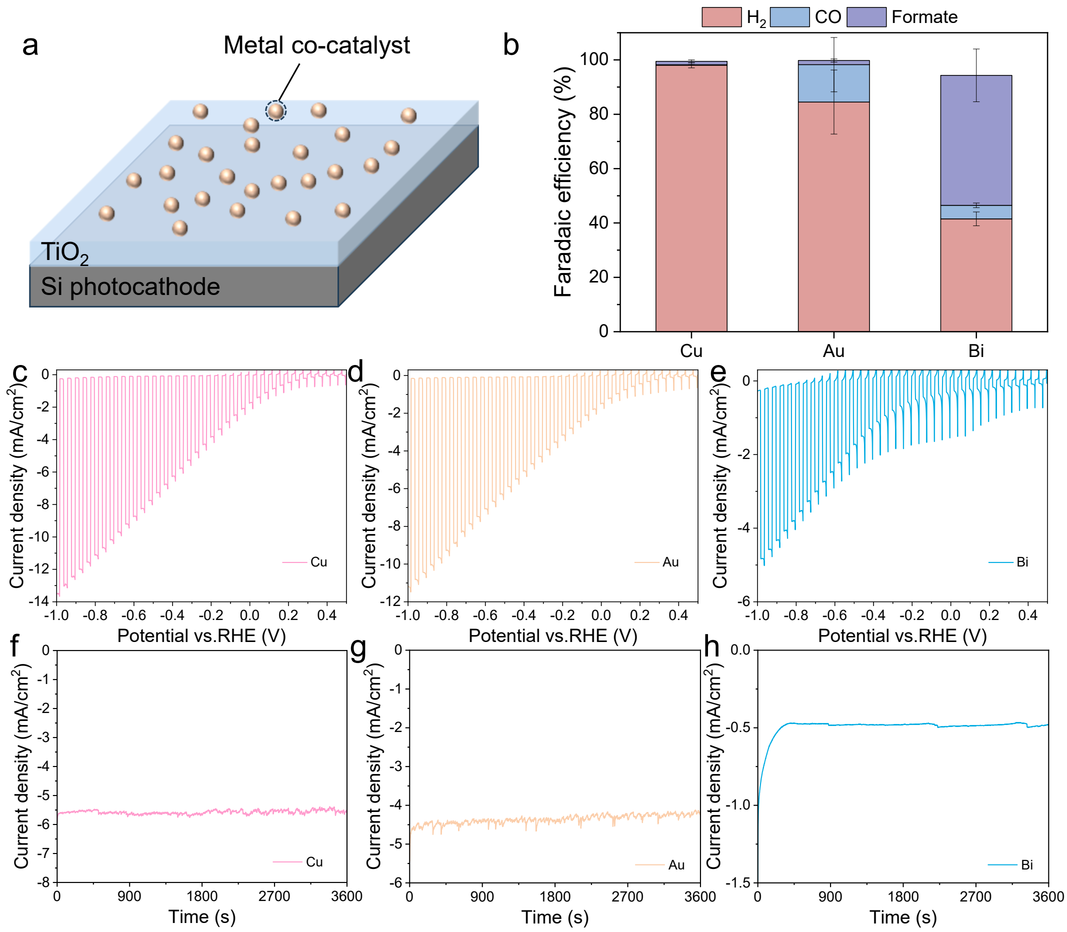

Figure S12. The schematic diagram and PEC performance of silicon photocathodes with Au/Cu/Bi as cocatalysts. a. The schematic diagram of silicon photocathodes with Au/Cu/Bi as cocatalysts. b. The Faradic efficiency of silicon photocathodes with Au/Cu/Bi as cocatalysts for PEC CO_2 reduction. c-h. The PEC CO_2 reduction performance of silicon photocathodes with Cu (c, f), Au (d, g), Bi (e, h) as cocatalysts.

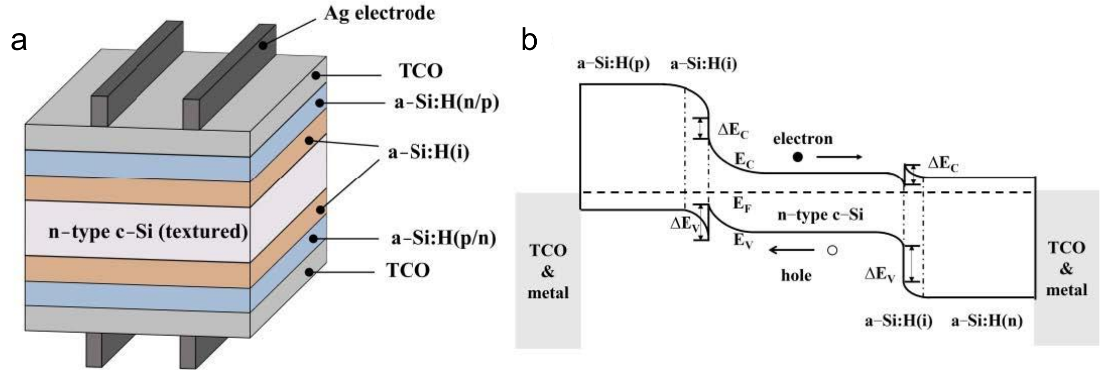

Figure S13. Schematic diagrams of the energy band structure of Si photocathodes. Reproduced with permission from Shi et al., Materials **16**, 3144 (2023). Copyright 2023 Authors, licensed under a Creative Commons Attribution (CC BY) License.[16]

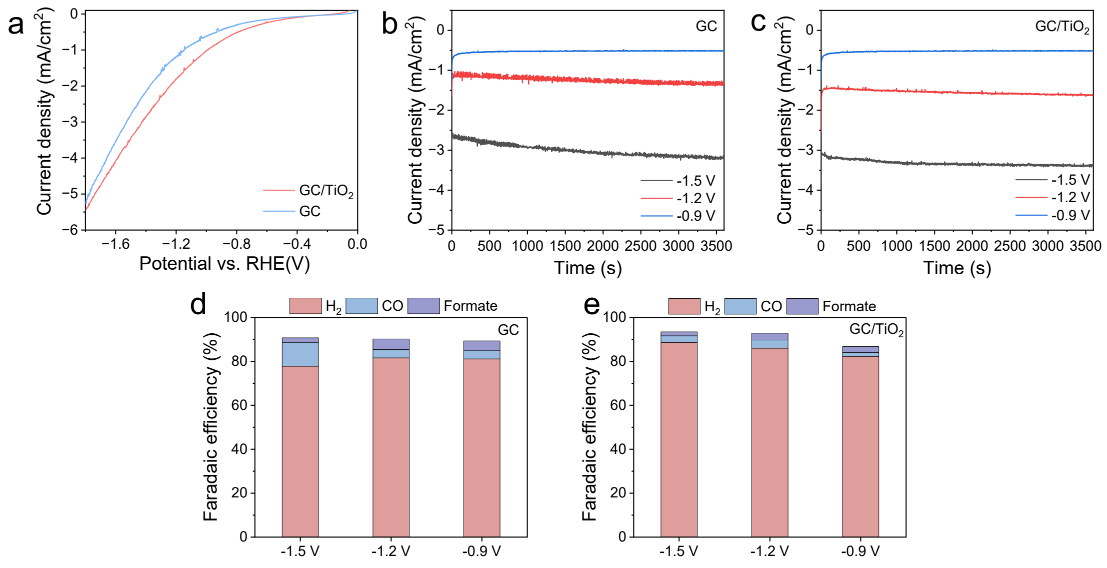

Figure S14. EC performance of glass carbon (GC) and glass carbon-TiO_2 (GC-TiO_2) for CO_2RR. (a) LSV curves of GC and GC-TiO_2. (b) Chronoamperogram of GC at varying potential. (c) Chronoamperogram of GC-TiO_2 at varying potential. (d) Faradaic efficiency of GC. (e) Faradaic efficiency of GC-TiO_2.

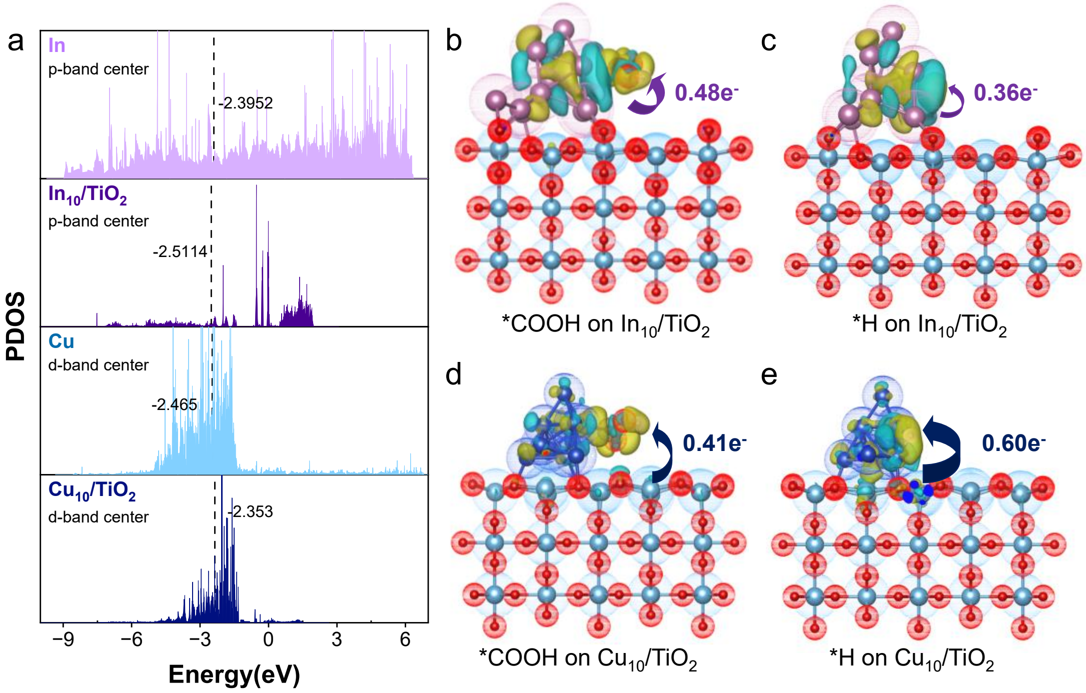

Figure S15. Electronic structure modulation of representative transition metal (Cu) and main-group metal (In) cocatalysts. (a) Projected density of states (PDOS) and calculated band centers relative to the Fermi level. (b–e) Charge density difference plots. The preferential charge injection into *COOH for In-based and *H for Cu-based systems.

For p-block elements (e.g., Sn, In, Bi), the p-band center determines the reaction pathway; specifically, a downward shift (moving away from the Fermi level) favors electron transfer to oxygen-bound intermediates (like *OCHO or *COOH), thereby facilitating formate generation. As displayed in Figure S15a, the In p-band center shifts downward from −2.3952 eV (isolated In) to −2.5114 eV (on TiO_2). Consistent with the mechanism observed in Bi/g-C_3N_4 systems where p-band downshifting enhances *OCHO adsorption[17], this electronic reconfiguration in In/TiO_2 is favorable for carboxylate intermediates. This is supported by the charge transfer analysis. Figure S15b shows that In/TiO_2 transfers significantly more electrons to *COOH (0.48 e^-) compared to *H (0.36 e^-, Figure S15c). This confirms that the p-band downshift effectively optimizes the orbital matching for COOH activation while suppressing competitive H binding.

For transition metals (Cu), general d-band theory suggests that an upward shift of the d-band center towards the Fermi level reduces the energy barrier for adsorption, thereby strengthening the bonding with key intermediates. As shown in Figure S15a, our PDOS calculations reveal that the Cu d-band center shifts upward from −2.465 eV (isolated Cu) to −2.353 eV (on TiO_2). Similar to the strain-induced d-band upshift observed in transition metal nanostructures, this upshift significantly enhances the surface reactivity. This enhanced reactivity is visually confirmed by the charge density difference maps. As depicted in Figure S15e, H* adsorption on Cu/TiO_2 induces a

substantial charge transfer of 0.60 e^-, which is significantly higher than the 0.41 e^- transferred to *COOH (Figure S15d). This result indicates that the d-band upshift creates a highly active surface with excessive H-affinity, thereby promoting HER.

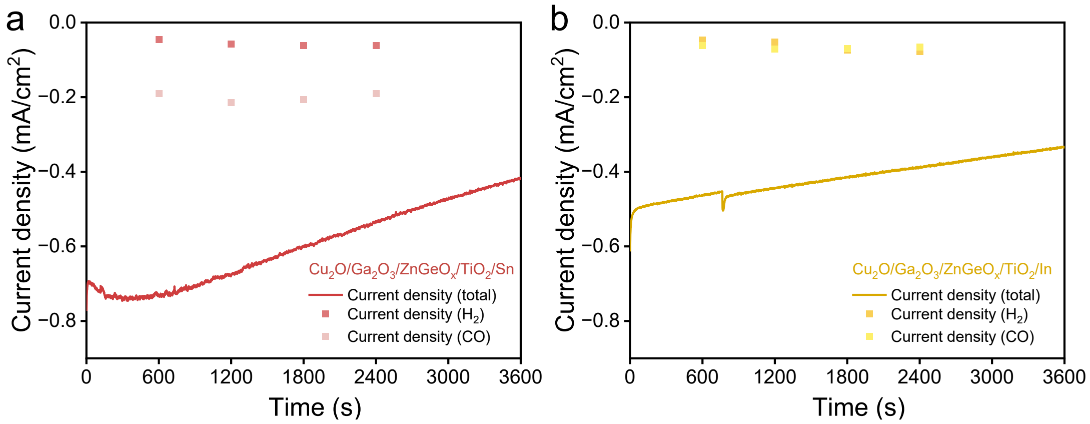

Figure S16. Partial current densities of H_2 and CO of (a) $Cu_2O/Ga_2O_3/ZnGeO_x/TiO_2/Sn$, (b) $Cu_2O/Ga_2O_3/ZnGeO_x/TiO_2/In$ at 0 V (versus RHE).

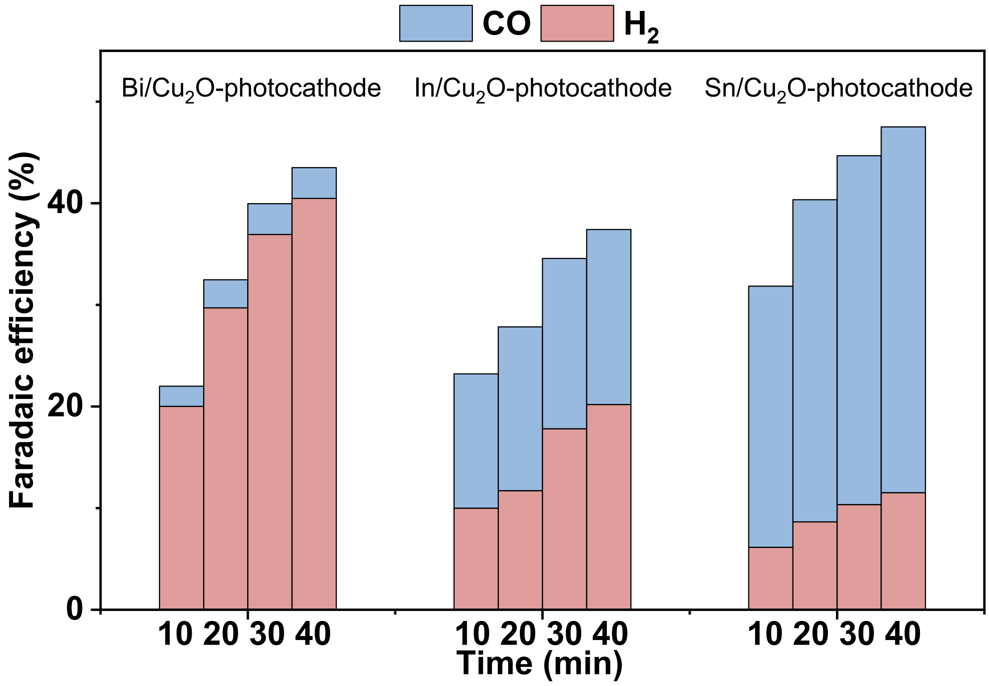

Figure S17. Gas products faradaic efficiency of Cu_2O photocathode with Bi/In/Sn as cocatalyst varying with testing time.

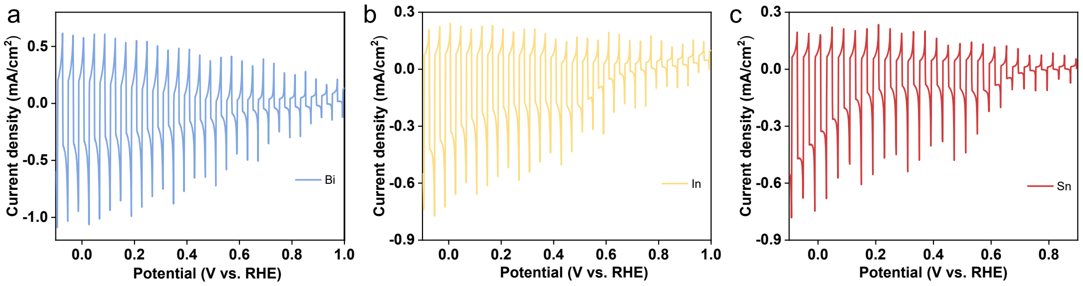

Figure S18. The linear sweep voltammetry (LSV) of Cu_2O photocathodes with Bi/In/Sn as cocatalyst after chronoamperogram measurement.

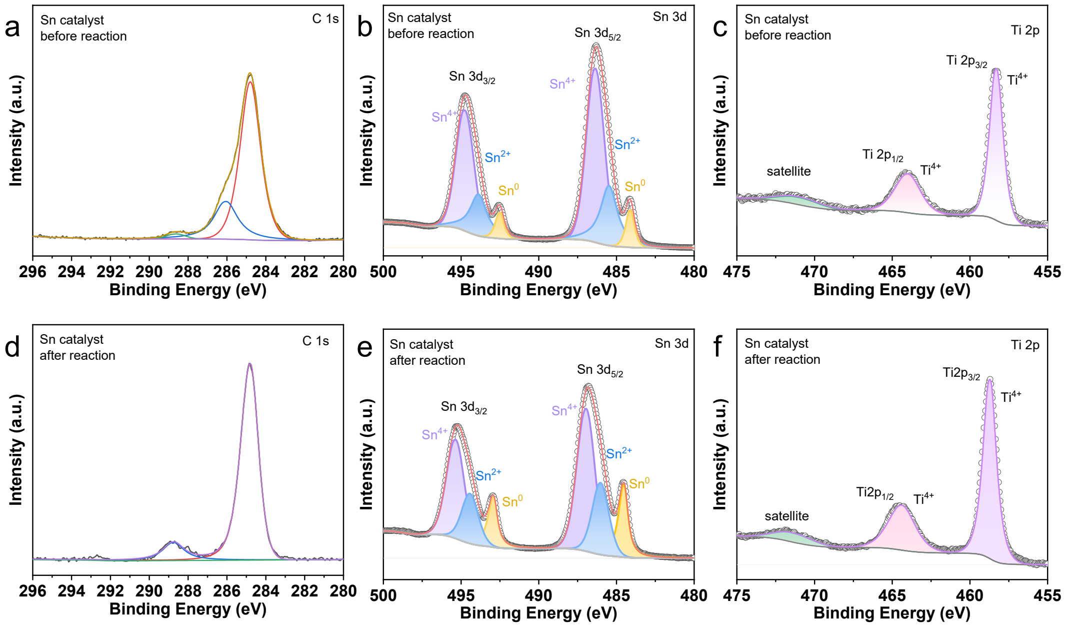

Figure S19. XPS spectra of the Cu_2O photocathodes with Sn as co-catalysts before (a, b, c) and after (d, e, f) PEC CO_2R test. (a, d) C 1s, (b, e) Sn 3d, (c, f) Ti 2p.

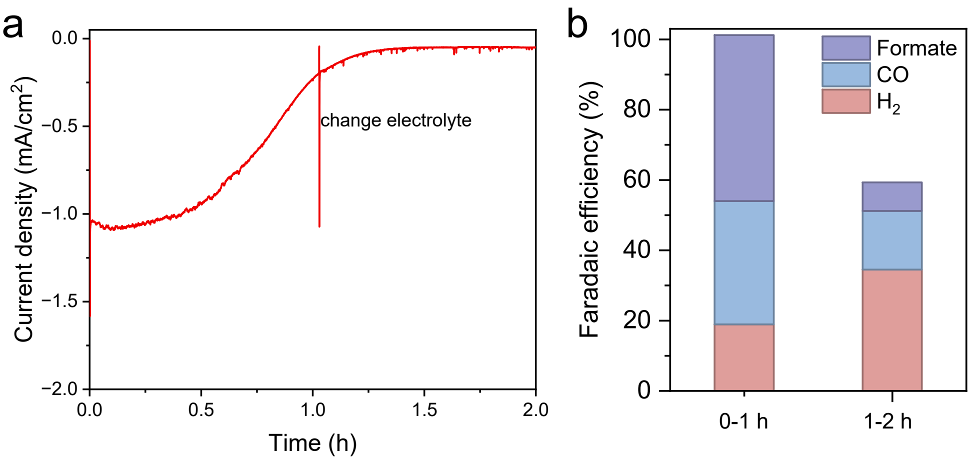

Figure S20. (a) Stability test for 2 h at 0 vs. RHE and (b) FE of $Cu_2O/Ga_2O_3/ZnGeO_x/TiO_2/Sn$ photocathodes.

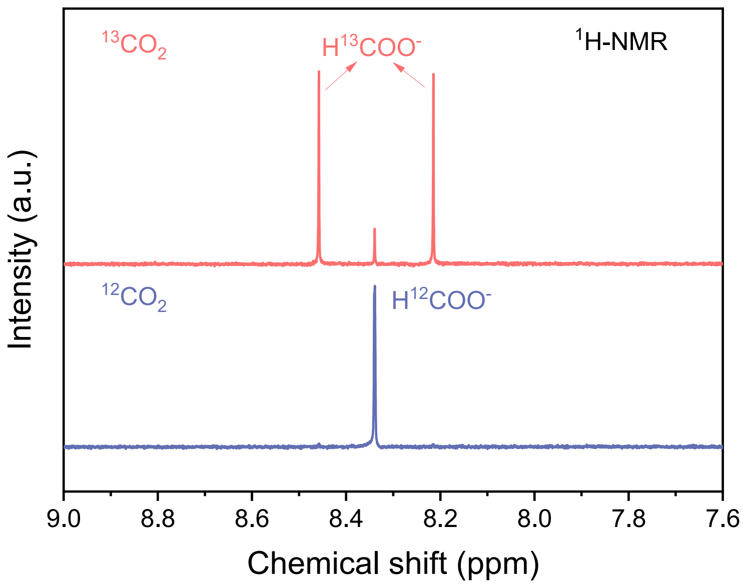

Figure S21. ^{1}H NMR identification of formate produced by 1 h PEC stability test of $Cu_2O/Ga_2O_3/ZnGeO_x/TiO_2/Sn$ photocathodes at 0 V (vs. RHE) under illumination in $^{13}CO_2$-saturated 0.1 M KOH solution and $^{12}CO_2$-saturated 0.1 M KOH solution. Small quantities of $H^{12}COO^-$ impurities may result from impurities of $^{12}CO_2$ in $^{13}CO_2$ gas.

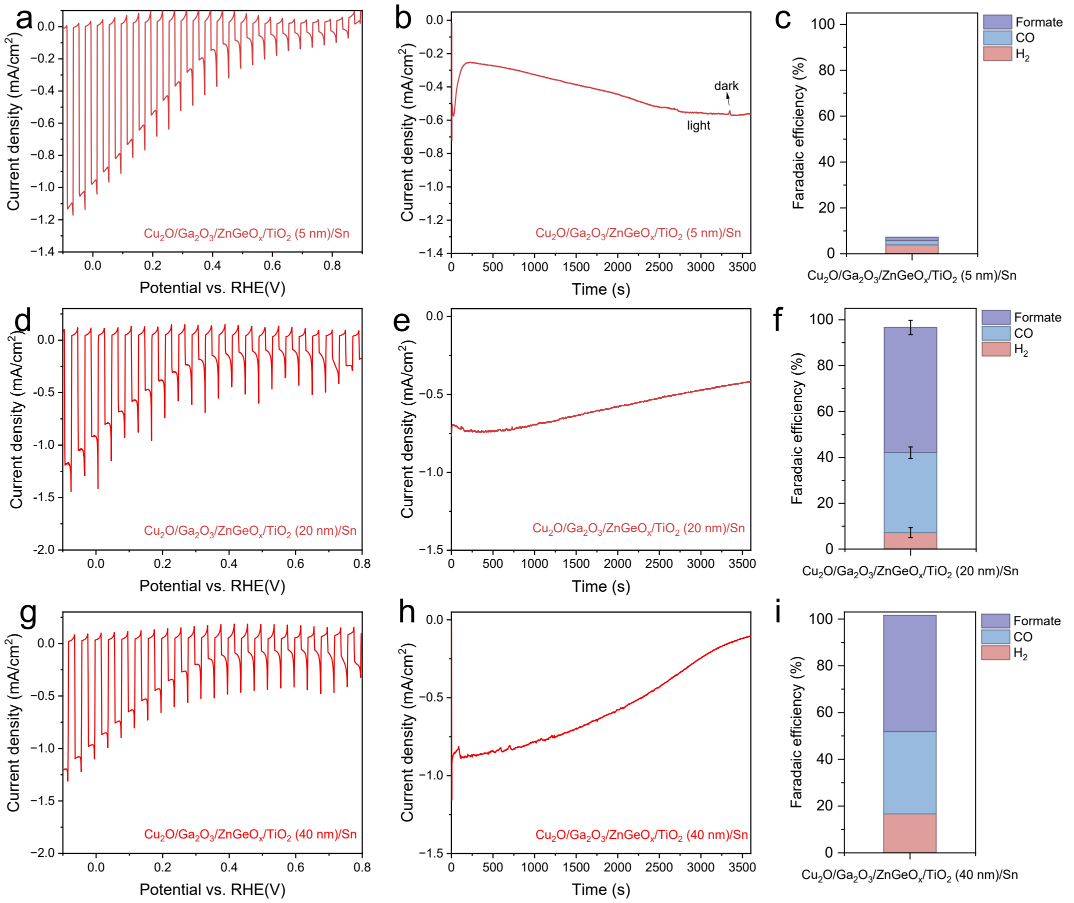

Figure S22. (a) LSV curves, (b) stability test and (c) FE of $Cu_2O/Ga_2O_3/ZnGeO_x/TiO_2$ (5 nm)/Sn photocathodes. (d) LSV curves, (e) stability test and (f) FE of $Cu_2O/Ga_2O_3/ZnGeO_x/TiO_2$ (20 nm)/Sn photocathodes. (g) LSV curves, (h) stability test and (i) FE of $Cu_2O/Ga_2O_3/ZnGeO_x/TiO_2$ (40 nm)/Sn photocathodes.

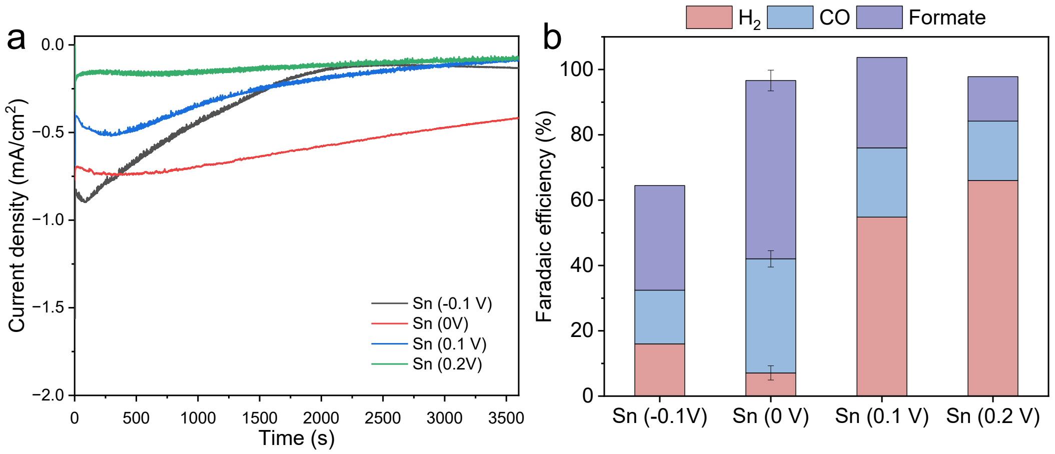

Figure S23. Chronoamperogram test (a) and FE (b) of $Cu_2O/Ga_2O_3/ZnGeO_x/TiO_2/Sn$ photocathodes at different potential under AM 1.5 G illumination.

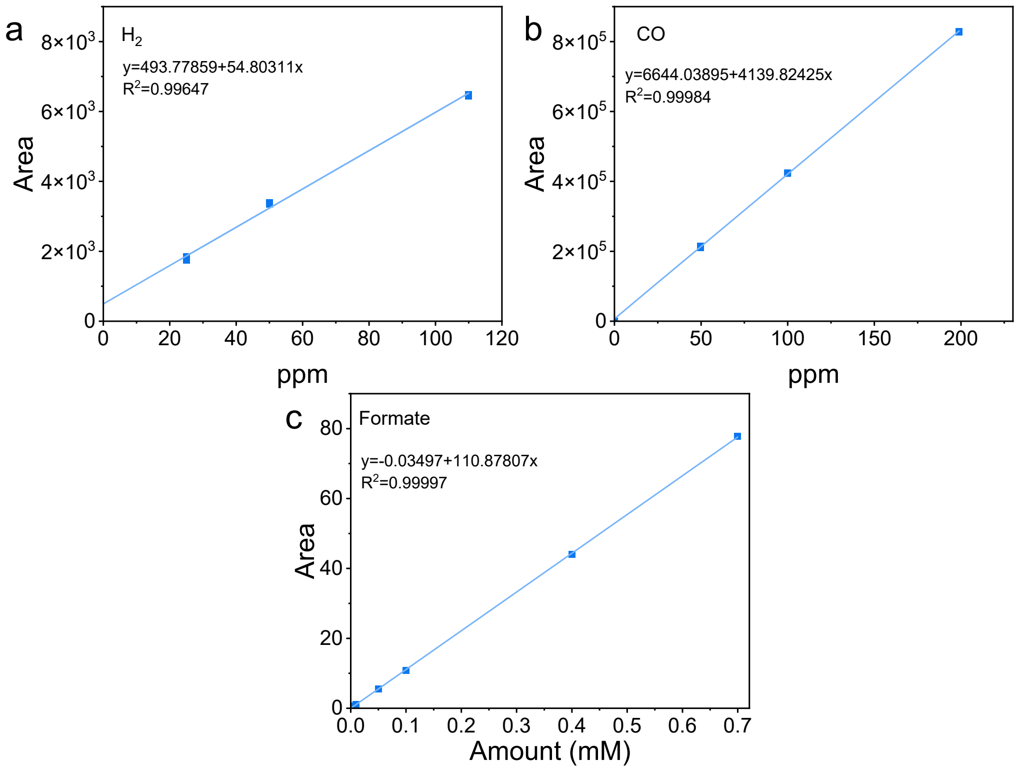

Figure S24. Calibration curves for product quantification. (a) H_2, (b) CO, (c) Formate.

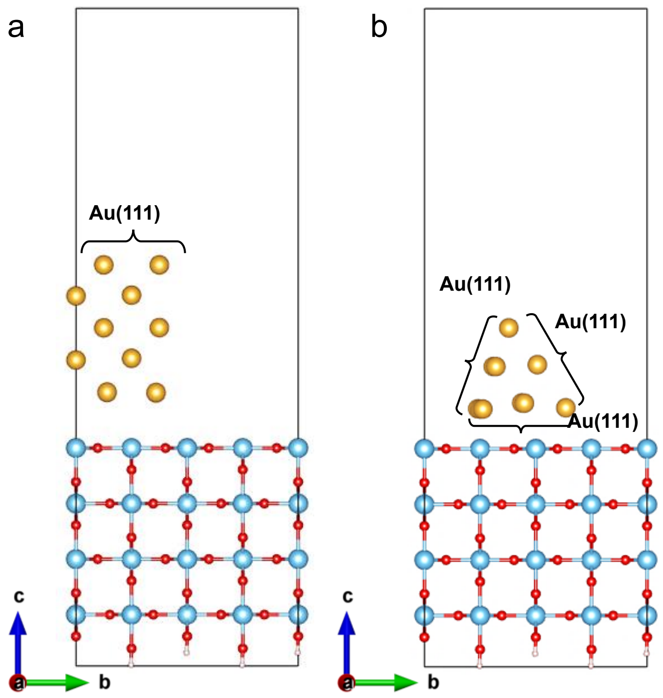

Figure S25. Different adsorption surfaces of plate stacking and cluster stacking. It should be noted that this diagram does not represent the final adsorption model; it is merely for comparison. (a) Two exposed adsorption surfaces when Au is deposited on the TiO_2 substrate in the form of a plate. (b) Three exposed adsorption surfaces when Au is deposited on the TiO_2 substrate in the form of a cluster of 10 atoms.

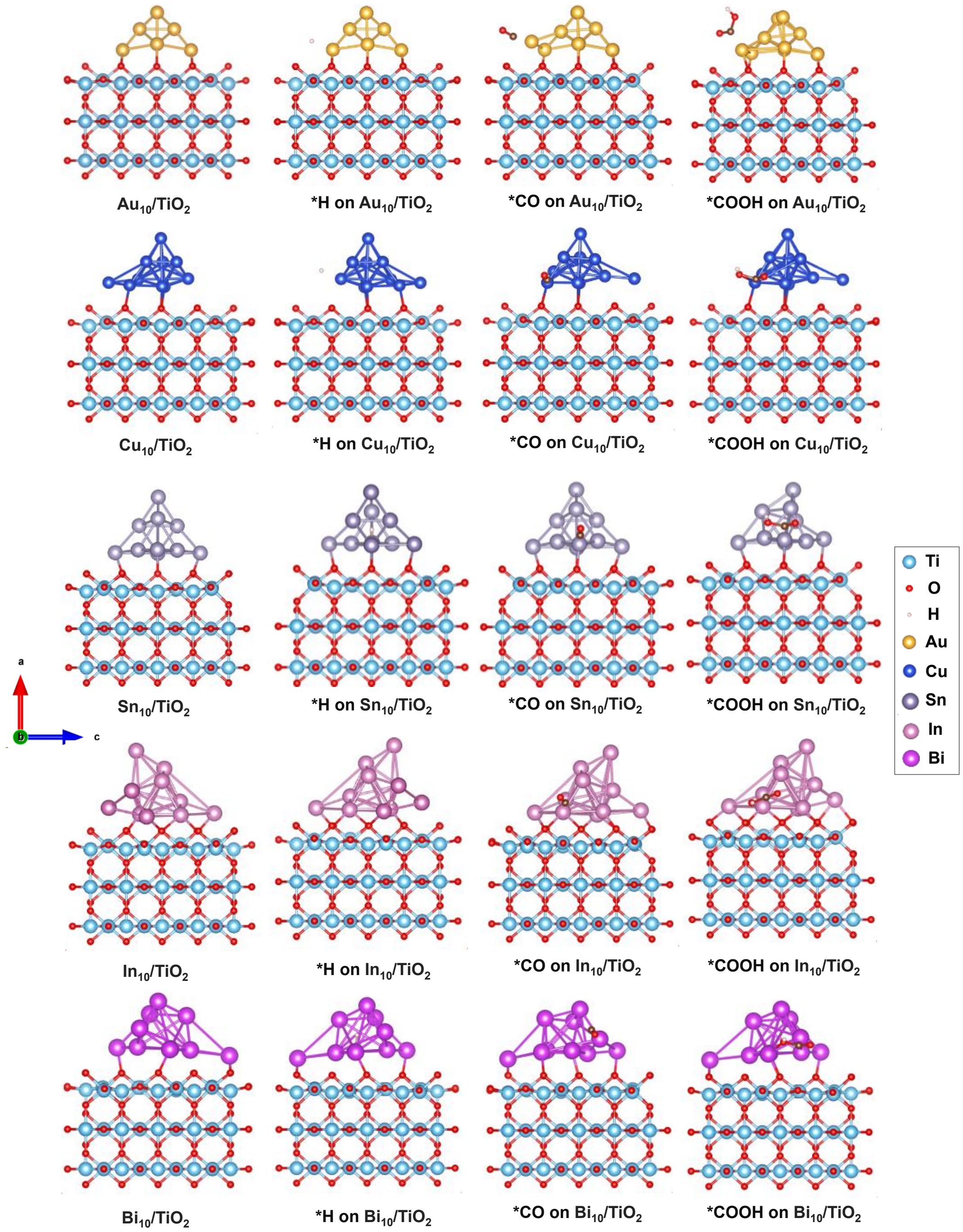

Figure S26. The optimized metal cluster/TiO_2 model demonstrated the capacity to adsorb the intermediates. The metal clusters in the model are arranged in a sequence from top to bottom, beginning with Au, then Cu, Sn, In, and finally Bi. From left to right, the adsorption of the model is as follows: non-adsorption, *H adsorption, *CO adsorption and *COOH adsorption.

Table S1. Variations in the adsorption energy (in eV) of COOH at cutoff energies of 500, 550, 600, and 700 eV on the Au_{10}/TiO_2 hybrid catalytic model. The case of 500 eV is chosen as the energy reference.

Cutoff (eV)	500	550	600	700
$\Delta E_{Au\_TiO2*COOH}$ (eV)	0	0.0220	-0.2168	-0.6210
ΔE_{Au_TiO2}(eV)	0	0.0238	-0.2064	-0.5950
$\Delta E_{COOH(g)}$ (eV)	0	-0.0021	-0.0103	-0.0242
ΔE(eV)	0	0.0003	0.0000	-0.0019
Δ	0%	-0.09%	0.01%	0.52%

Table S2. Comparison of the energy (in eV) of different adsorption sites of COOH on pure metal Au, the top adsorption is chosen as energy reference.

Site	top	bridge	hcp	fcc
ΔE	0	0.7949	0.8437	0.6264

Table S3. Performance comparison of recent reports (2023-2025) about photocathode for PEC CO_2RR.

Photocathode	J_{HCOO^-} (mA cm^{-2}) (potential/V)	J (mA cm^{-2}) (potential/V)	FE $_{HCOO^-}$(%) (potential/V)	electrolyte	Ref
Bi/GaN/Si	～3.4 (-0.2)	～4 (-0.2)	85.2 (-0.2)	0.5 $KHCO_3$	[18]
FeOOH/p-nCu_2O/Co:CdS	2.2 (-0.75)	2.7 (-0.75)	82.9 (-0.75)	0.5 $KHCO_3$	[19]
Bi-Sn/SiNWs	～4.4 (-1.02)	～5 (-1.02)	88.67 (-1.02)	0.1 $KHCO_3$	[20]
Si−Bi@C800	9.3 (-0.9)	10.19 (-0.9)	91.23 (-0.9)	0.1 $KHCO_3$	[21]
CuBi-300/Si	5.2 (-0.3)	5.4 (-0.3)	95 (-0.3)	0.5 $KHCO_3$	[22]
S-Bi/SiNWs	4.6 (-0.8)	～5 (-0.8)	92.3 (-0.8)	0.1 $KHCO_3$	[23]
$Cu_2O/Ga_2O_3/TiO_2$/Sn/SnO_x	0.4 (-0.8)	1.18 (-0.8)	34.35 (+0.24)	0.5 $KHCO_3$	[24]
$Cu_2O/Ga_2O_3/TiO_2$/In	0.99 (−0.9 V vs.Ag/AgCl)	1.6 (−0.9 V vs.Ag/AgCl)	62.1 (−0.9 V vs.Ag/AgCl)	0.1 $KHCO_3$	[25]
$Cu_2O/Ga_2O_3/TiO_2$/SnO_x	1.2 (-0.1 V)	1.6 (-0.1 V)	75.4 (-0.1)	1 $KHCO_3$	[26]
$Cu_2O/Ga_2O_3/TiO_2$/Bi	0.284 (0 V)	0.44 (0 V)	64.7 (0)	0.1 $KHCO_3$	This work
$Cu_2O/Ga_2O_3/TiO_2$/In	0.348 (0 V)	0.55 (0 V)	63.2 (0)	0.1 $KHCO_3$	This work
$Cu_2O/Ga_2O_3/TiO_2$/Sn	0.37 (0 V)	0.68 (0 V)	54.6 (0)	0.1 $KHCO_3$	This work

Reference

1. Kresse G and Joubert D. From ultrasoft pseudopotentials to the projector augmented-wave method. *Phys Rev B* 1999; **59**: 1758.

2. Kresse G and Furthmüller J. Efficiency of ab-initio total energy calculations for metals and semiconductors using a plane-wave basis set. *Comput Mater Sci* 1996; **6**: 15-50.

3. Kresse G and Furthmüller J. Efficient iterative schemes for ab initio total-energy calculations using a plane-wave basis set. *Phys Rev B* 1996; **54**: 11169.

4. Hammer B, Hansen L B and Nørskov J K. Improved adsorption energetics within density-functional theory using revised Perdew-Burke-Ernzerhof functionals. *Phys Rev B* 1999; **59**: 7413.

5. Grimme S, Antony J and Ehrlich S *et al.* A consistent and accurate ab initio parametrization of density functional dispersion correction (DFT-D) for the 94 elements H-Pu. *J Chem Phys* 2010; **132**: 154104.

6. Liu F, Gao X and Shi R *et al.* Concerted and Selective Electrooxidation of Polyethylene-Terephthalate-Derived Alcohol to Glycolic Acid at an Industry-Level Current Density over a Pd−Ni(OH)$_2$ Catalyst. *Angew Chem Int Ed* 2023; **62**: e202300094.

7. Guo Y, Wang M and Zhu Q *et al.* Ensemble effect for single-atom, small cluster and nanoparticle catalysts. *Nat Catal* 2022; **5**: 766-76.

8. Garlyyev B, Fichtner J and Piqué O *et al.* Revealing the nature of active sites in electrocatalysis. *Chem Sci* 2019; **10**: 8060-75.

9. Vogt C and Weckhuysen B M. The concept of active site in heterogeneous catalysis. *Nat Rev Chem* 2022; **6**: 89-111.

10. Zhang Z, Wang S-S and Song R *et al.* The most active Cu facet for low-temperature water gas shift reaction. *Nat Commun* 2017; **8**: 488.

11. Linsebigler A L, Lu G and Yates J T, Jr. Photocatalysis on TiO$_2$ Surfaces: Principles, Mechanisms, and Selected Results. *Chem Rev* 1995; **95**: 735-58.

12. Burnside S D, Shklover V and Barbé C *et al.* Self-Organization of TiO$_2$ Nanoparticles in Thin Films. *Chem Mater* 1998; **10**: 2419-25.

13. Labat F, Baranek P and Adamo C. Structural and Electronic Properties of Selected Rutile and Anatase TiO$_2$ Surfaces: An ab Initio Investigation. *J Chem Theory Comput* 2008; **4**: 341-52.

14. Zhu H-Q and Feng Q. Microscopic characteristics mechanism of optical gas sensing material rutile titanium dioxide (110) surface adsorption of CO molecules. *Acta Phys Sin* 2014; **63**: 133101.

15. Cheng J, Wu L and Luo J. Improving the photovoltage of Cu$_2$O photocathodes with dual buffer layers. *Nat Commun* 2023; **14**: 7228.

16. Shi C, Shi J and Guan Z *et al.* Surface Cleaning and Passivation Technologies for the Fabrication of High-Efficiency Silicon Heterojunction Solar Cells. *Materials* 2023; **16**: 3144.

17. Wang S, Yang Y and Zhao Y *et al.* Electronic Modulation of Bismuth by g-C$_3$N$_4$ Constructs Electron-Enriched Active Sites for Accelerated CO$_2$ Electroreduction to Formate. *ACS Appl Mater Interfaces* 2025; **17**: 28234-43.

18. Pan Y, Zhang H and Zhang B *et al.* Renewable formate from sunlight, biomass and carbon dioxide in a photoelectrochemical cell. *Nat Commun* 2023; **14**: 1013.
19. Liu S, Guo Z and Yang Y *et al.* Cobalt-doped CdS quantum dots enhanced photoelectroreduction of CO_2 to formic acid with high selectivity. *Environ Chem Lett.* 2024; **22**: 463-70.
20. Shen W, Yang Z and Wang J *et al.* Bi–Sn Co-Catalyst-Modified p-Si Nanowire Array Photocathodes for Photoelectrocatalytic CO_2 Reduction to Formate. *ACS Sustainable Chem Eng* 2023; **11**: 13451-7.
21. Chen Y, Kang J and Zou M *et al.* Porous Carbon Nanorods Encapsulating Bismuth Nanoparticles Promote p-Si Nanowire Array for Photoelectrocatalytic CO_2 Reduction to Formate. *Ind Eng Chem Res* 2024; **63**: 21831-40.
22. Li W, Hong J and Shang J *et al.* In situ construction of CuBi-MOF derived heterojunctions with electron-rich effects enhances localized CO_2 enrichment integrated with Si photocathodes for CO_2 reduction. *Appl Catal B: Environ* 2025; **365**: 124890.
23. Ma A, Lee Y and Seo D *et al.* Unlocking the Potential of Bi_2S_3-Derived Bi Nanoplates: Enhanced Catalytic Activity and Selectivity in Electrochemical and Photoelectrochemical CO_2 Reduction to Formate. *Adv Sci* 2024; **11**: 2400874.
24. Xia M, Pan L and Liu Y *et al.* Efficient Cu_2O Photocathodes for Aqueous Photoelectrochemical CO_2 Reduction to Formate and Syngas. *J Am Chem Soc* 2023; **145**: 27939-49.
25. Liu Y, Chen B and Liu Y *et al.* Photoelectrochemical CO_2 reduction to formic acid using as cuprous oxide-based photocathodes. *Fuel* 2025; **387**: 134168.
26. Wan L, Wu L and Han Y *et al.* Activating SnO_x-Coated Cu_2O Photocathodes for Efficient Photoelectrochemical CO_2 Reduction and Unassisted Tandem Device Integration. *J Mater Chem A* 2026; 14: 2795-803.
